# Sperm miR‐142‐3p Reprogramming Mediates Paternal Pre‐Pregnancy Caffeine Exposure‐Induced Non‐Alcoholic Steatohepatitis in Male Offspring Rats

**DOI:** 10.1002/advs.202405592

**Published:** 2024-09-18

**Authors:** Cong Zhang, Yu Guo, Yi Liu, Kexin Liu, Wen Hu, Hui Wang

**Affiliations:** ^1^ Department of Pharmacology School of Basic Medical Sciences Wuhan University Wuhan 430071 China; ^2^ Hubei Provincial Key Laboratory of Developmentally Originated Disease Wuhan 430071 China; ^3^ Department of Pharmacy Zhongnan Hospital of Wuhan University Wuhan 430072 China

**Keywords:** ACSL4, miR‐142‐3p, non‐alcoholic steatohepatitis, paternal pre‐pregnant caffeine exposure, sperm reprogramming

## Abstract

Numerous studies have suggested a strong association between paternal adverse environmental exposure and increased disease susceptibility in offspring. However, the impact of paternal pre‐pregnant caffeine exposure (PPCE) on offspring health remains unexplored. This study elucidates the sperm reprogramming mechanism and potential intervention targets for PPCE‐induced non‐alcoholic steatohepatitis (NASH) in offspring. Here, male rats are administrated caffeine (15–60 mg kg^−1^/d) by gavage for 8 weeks and then mated with females to produce offspring. This study finds that NASH with transgenerational inheritance occurred in PPCE adult offspring. Mechanistically, a reduction of miR‐142‐3p is implicated in the occurrence of NASH, characterized by hepatic lipid metabolism dysfunction and chronic inflammation through an increase in ACSL4. Conversely, overexpression of miR‐142‐3p mitigated these manifestations. The origin of reduced miR‐142‐3p levels is traced to hypermethylation in the miR‐142‐3p promoter region of parental sperm, induced by elevated corticosterone levels rather than by caffeine per se. Similar outcomes are confirmed in offspring conceived via in vitro fertilization using miR‐142‐3p^KO^ sperm. Overall, this study provides the first evidence of transgenerational inheritance of NASH in PPCE offspring and identifies miR‐142‐3p as a potential therapeutic target for NASH induced by paternal environmental adversities.

## Graphical abstract

The study provides the pioneering demonstration of the link between paternal pre‐pregnant caffeine exposure (PPCE) and disrupted hepatic lipid metabolism in male offspring rats, leading to the development of NASH in adulthood. The underlying mechanism involves elevated glucocorticoid levels from PPCE, which induce hypermethylation in the sperm miR‐142‐3p promoter region and mediated liver lipid metabolism disorders in male offspring rats.

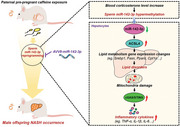

## 1 Introduction

Non‐alcoholic fatty liver disease (NAFLD) has become the world's most prevalent chronic liver metabolic disease, affecting ≈30% of the global population.^[^ ^1^ ^]^ Nonalcoholic steatohepatitis (NASH) represents a severe progressive form of NAFLD. It is understood that the pathogenesis of NAFLD involves a complex interplay among genetic susceptibility, environmental factors, and metabolic stress.^[^ ^2^ ^]^ According to the “Developmental Origins of Health and Disease (DOHaD)” theory, adult metabolic diseases such as NAFLD may originate during the fetal period,^[^ ^3^ ^]^ whereby adverse environmental factors during embryonic development can lead to metabolic adaptations and program‐lasting changes in the offspring, increasing their susceptibility to various metabolic diseases post‐birth.^[^ ^4^, ^5^, ^6^ ^]^ Compared to embryos, gametes (sperm and oocytes) are more susceptible to adverse environments during their prolonged development and maturation period.^[^ ^7^ ^]^ Paternal Origins of Health and Disease (POHaD) theory has emerged from extensive research, highlighting paternal factors—such as adverse lifestyle and environmental exposures, as significant independent risk factors for abnormal development and diseases in the offspring.^[^ ^8^, ^9^ ^]^ Epidemiological data have demonstrated a markedly increased risk of obesity and diabetes in individuals whose fathers experienced famine.^[^ ^10^ ^]^ Furthermore, clinical and laboratory studies have identified that paternal pre‐pregnant adverse environmental exposures, such as smoking, high‐fat diet, and chronic stress, can cause intrauterine growth retardation (IUGR) in fetuses and postnatal lipid metabolism dysfunction in offspring.^[^ ^11^, ^12^, ^13^, ^14^ ^]^ This evidence underscores the significant role of the paternal pre‐pregnancy environment in predisposing offspring to multiple diseases, including NAFLD.

Long‐term exposure to adverse events, such as xenobiotics and psychological stress, induces chronic stress and may cause various diseases.^[^ ^15^ ^]^ Caffeine, a central stimulant found in many beverages, including coffee, tea, chocolate, and soft drinks, acts as a chronic stressor with reproductive and developmental toxicity.^[^ ^16^ ^]^ Our previous studies have confirmed the detrimental long‐term effects of maternal caffeine consumption during pregnancy on offspring health.^[^ ^17^, ^18^, ^19^, ^20^ ^]^ However, male caffeine consumption during reproductive years often exceeds that of females,^[^ ^21^, ^22^ ^]^ highlighting the need for increased attention to the potential risks of paternal caffeine intake on progeny health. Research has shown that paternal pre‐pregnant caffeine intake can impair early brain development in offspring, associated with increased paternal glucocorticoid levels.^[^ ^23^, ^24^ ^]^ Glucocorticoids are crucial in germ cell formation, embryonic development, and postnatal health.^[^ ^25^ ^]^ These findings have prompted further investigation into the impact of paternal caffeine exposure and the resultant high glucocorticoid levels on the metabolic health of their offspring.

Adverse environmental exposure before mating alters the epigenetic modification of sperm, such as DNA methylation and non‐coding RNA. These “marks” are transmitted to offspring through sperm, influencing developmental programming and progeny phenotypes across multiple generations.^[^ ^26^, ^27^ ^]^ MicroRNAs (miRNAs), as critical non‐coding RNAs, are involved in various cellular processes related to gametogenesis and embryonic development.^[^ ^28^, ^29^ ^]^ Clinical and experimental studies have shown that sperm miRNAs, acting as marker and regulators of epigenetic modifications for key genes, carry paternal epigenetic information and transmit it to offspring, impacting their long‐term health.^[^ ^30^, ^31^ ^]^ Adverse paternal pre‐pregnancy lifestyle and chronic stress affect the expression of multiple sperm miRNAs and post‐transcription levels of their downstream target genes, which mediate hepatic lipid metabolism dysfunction in offspring.^[^ ^32^, ^33^ ^]^ Furthermore, changes in sperm miRNA expression are associated with increased glucocorticoids under paternal chronic stress.^[^ ^32^, ^34^ ^]^ Given these findings, a thorough investigation into the effects of elevated paternal glucocorticoids on sperm miRNA expression and the long‐term health of offspring is warranted.

In this study, a paternal pre‐pregnant caffeine exposure (PPCE) rat model was established to simulate caffeine‐induced chronic stress in humans and to explore the developmental origins of paternal stress‐driven NASH in offspring.

## 2 Results

### 2.1 PPCE Causes Adult NASH in Male Offspring

**Figure 1:**
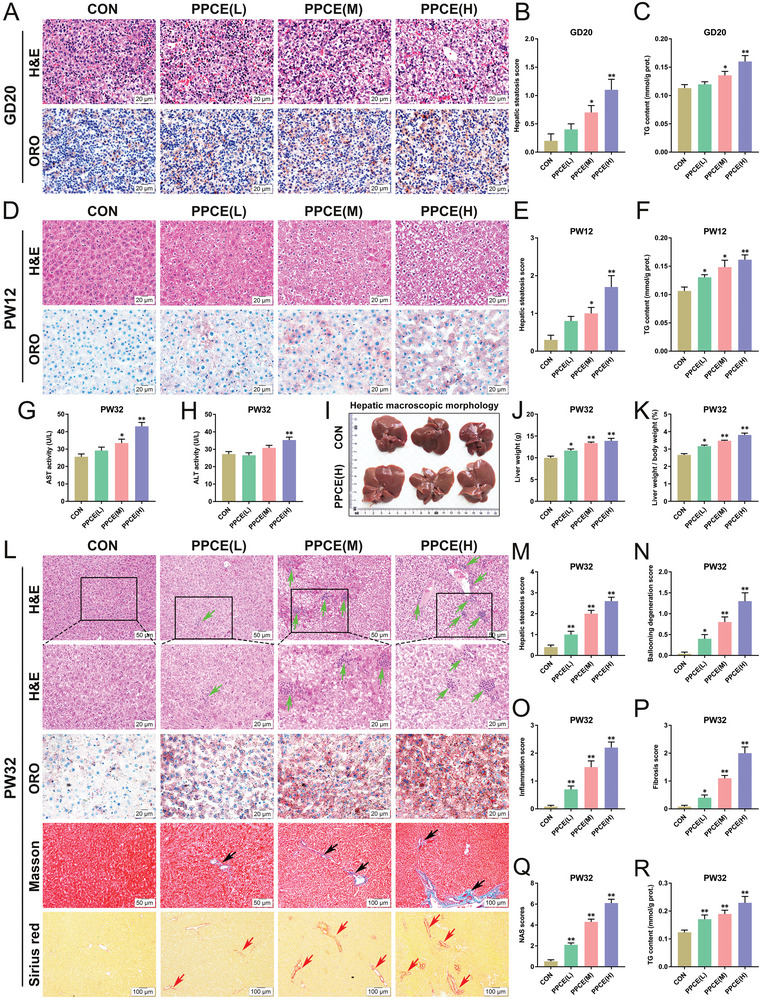
PPCE causes adult NASH in male offspring. A,B) Liver H&E and ORO staining at GD20 and PW12 (400×); C,D) Liver steatosis score at GD20 and PW12; E,F) Liver TG contents at GD20 and PW12; G,H) Serum AST and ALT activities at PW32; I) Liver appearance morphology at PW32; J,K) Liver weight and ratio of liver weight to body weight at PW32; L) Liver H&E, ORO, Masson staining, and Sirius red staining at PW32 (200×, 400×); M–Q) NAS score and fibrosis score; R) Liver TG content at PW32. Mean ± S.E.M., *n* = 12. ^*^ *P* < 0.05, ^**^ *P* < 0.01 versus CON group (two‐tailed *t*‐test). Green arrow: inflammatory infiltration. Black and red arrows: deposition of collagen fibers.

To confirm that paternal caffeine intake can induce NASH in adult offspring, a PPCE rat model was established. First, we assessed the effects of PPCE on liver pathology, NAFLD activity score (NAS), and triglyceride metabolism in male offspring before and after birth. Pathological analyses revealed significant fat vacuole‐like degeneration and lipid accumulation in the livers of the PPCE male offspring on GD20 and PW12 (**Figure** **1**A,B), along with marked increases in hepatic steatosis scores (Figure 1C,D) and triglyceride content (Figure 1E,F). By PW32, serum transaminase activities (AST and ALT) had significantly risen in the PPCE group (Figure 1G,H), and the liver exhibited a light brown appearance with significant increases in liver weight and liver index (Figure 1I–K). H&E, Masson, and Sirius red staining showed typical histological features of NASH in the liver of PPCE male offspring at PW32, including pronounced vacuolar‐like steatosis, inflammatory infiltration (green arrows), and collagen fiber deposition (black and red arrows) (Figure 1L). Additionally, liver NAS and triglyceride content showed significant increases (Figure 1M–R). It is suggested that PPCE leads to hepatic lipid accumulation in male offspring and the occurrence of NASH in adulthood.

This study also revealed that while PPCE induced NAFLD in female offspring during adulthood, the liver pathological changes were less severe than in males, with no significant inflammatory infiltration or fibrosis observed (Figure S1, Supporting Information). In summary, PPCE can induce adult NASH in the offspring, displaying notable gender differences, particularly in males. Therefore, we focused on the programming mechanisms by which PPCE led to the occurrence of NASH in male offspring in the subsequent study.

### 2.2 PPCE Induces Hepatic Lipid Metabolism Disorders and Chronic Inflammatory Activation in Male Offspring Before and After Birth

**Figure 2:**
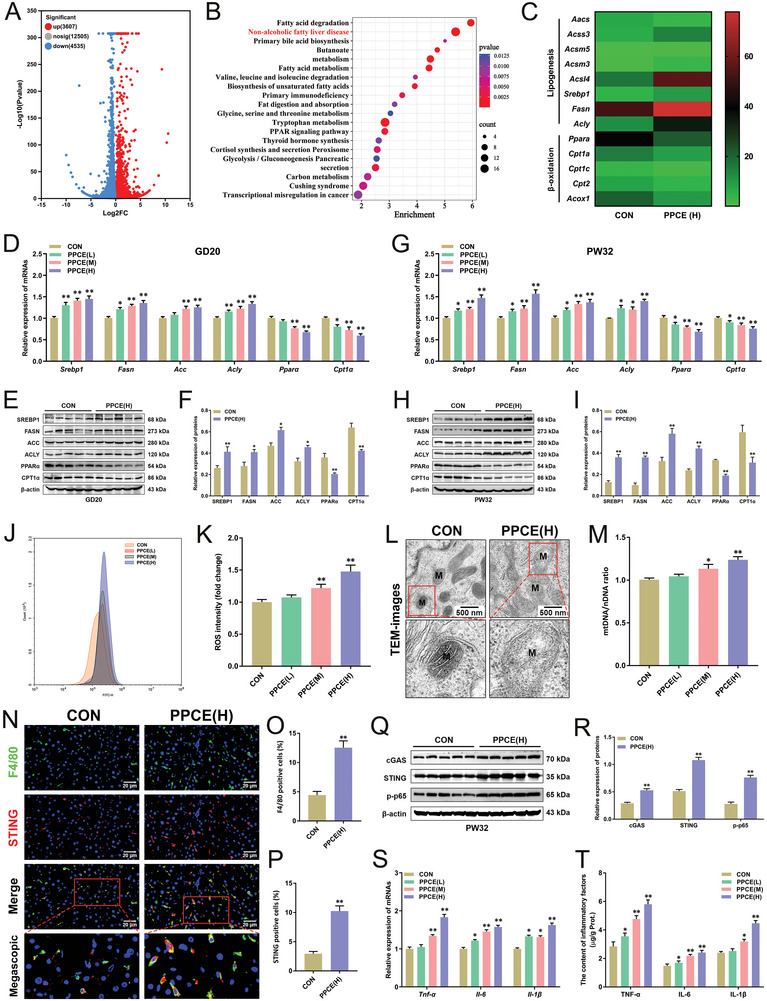
PPCE induces hepatic lipid metabolism disorders and chronic inflammatory activation in male offspring before and after birth. A) Volcanogram of differentially expressed genes at GD20; B) KEGG analysis at GD20; C) Differentially expressed lipid metabolic genes at GD20; D) The mRNA expression of *Srebp1*, *Fasn*, *Acc*, *Acly*, *Pparα*, and *Cpt1α* at GD20 (*n =* 12); E,F) Representative immunoblots and quantitative analysis of SREBP1, FASN, ACC, ACLY, PPAR*α*, and CPT1*α* protein GD20 (*n =* 5); G) The mRNA expression of *Srebp1*, *Fasn*, *Acc*, *Acly*, *Pparα*, and *Cpt1α* at PW32 (*n =* 12); H,I) Representative immunoblots and quantitative analysis of SREBP1, FASN, ACC, ACLY, PPAR*α*, and CPT1*α* protein at PW32 (*n =* 5); J,K) Liver ROS levels at PW32 (*n =* 12); L) TEM images at PW32; M) mtDNA copy numbers at PW32 (*n =* 12); N–P) Typical immunofluorescence images and statistical analysis of fluorescence intensity for F4/80 and STING at PW32; Q,R) Representative immunoblots and quantitative analysis of cGAS, STING, and p‐p65 protein at PW32 (*n =* 5); S,T) The mRNA expression and contents of TNF‐*α*, IL‐6, and IL‐1*β* at PW32 (*n =* 12). Mean ± S.E.M. ^*^ *P <* 0.05, ^**^ *P <* 0.01 versus CON group (two‐tailed *t*‐test).

Further examination of hepatic lipid metabolism and inflammatory response in PPCE male offspring rats before and after birth was conducted. RNA‐seq analysis revealed significant gene expression changes in the liver of fetal rats in the PPCE group compared to the CON group (**Figure** **2**A). In the liver of PPCE fetal rats, KEGG functional enrichment analysis revealed that these differentially expressed genes were primarily linked to lipid metabolic signaling, critical in NAFLD development (Figure 2B). Specifically, genes related to lipid synthesis were upregulated while those associated with *β*‐oxidation were downregulated (Figure 2C). Key factors in hepatic lipid synthesis and *β*‐oxidation were further verified using RT‐qPCR and Western blot. In both GD20 and PW32, the mRNA and protein levels of key transcription factors and functional enzymes (such as SREBP1, FASN, ACC, and ACLY) involved in hepatic lipogenesis were significantly higher in the PPCE group, while those for fatty acid *β*‐oxidation (such as PPAR*α* and CPT1*α*) were significantly lower (Figure 2D–I). These results suggested that PPCE could enhance hepatic fatty acid synthesis and reduce *β*‐oxidation in male offspring rats before and after birth.

Lipotoxicity, resulting from excessive lipid accumulation, can induce mitochondria damage and elevated reactive oxygen species (ROS), thereby promoting inflammatory responses in hepatocytes and NAFLD progression.^[^ ^35^ ^]^ Changes in mitochondrial function and inflammatory response in hepatocytes at PW32 were also assessed. Hepatic ROS levels were significantly higher in the PPCE group (Figure 2J,K), with severe mitochondrial damage observed (mitochondria swelling, rupture, and loss of mitochondrial cristae) (Figure 2L), and a significant increase in mtDNA copies (Figure 2M). This indicates that PPCE could cause mitochondrial damage and oxidative stress in the hepatocytes of male offspring. The release of relevant molecular patterns such as mtDNA from this oxidative stress injury can activate STING‐mediated inflammatory responses.^[^ ^36^ ^]^ Examination of the STING‐NF‐κB inflammatory signaling in the liver showed that macrophages in the PPCE group were activated and STING expression in macrophages was significantly increased (Figure 2N–P). Additionally, the protein expressions of cGAS, STING, and p‐NF‐κB p65 (p‐p65) and the mRNA levels and content of pro‐inflammatory factors (TNF‐*α*, IL‐6, and IL‐1*β*) were significantly increased (Figure 2Q–T). STING‐related inflammation was also observed in the fetal liver of PPCE male offspring (Figure S2, Supporting Information). These results indicated that PPCE can cause hepatic lipid metabolism dysfunction, lipid accumulation, and chronic inflammation in male offspring before and after birth.

### 2.3 miR‐142‐3p is a Critical Target Mediating PPCE‐Induced Hepatic Lipid Metabolism Disorders and Chronic Inflammation in Male Offspring

**Figure 3:**
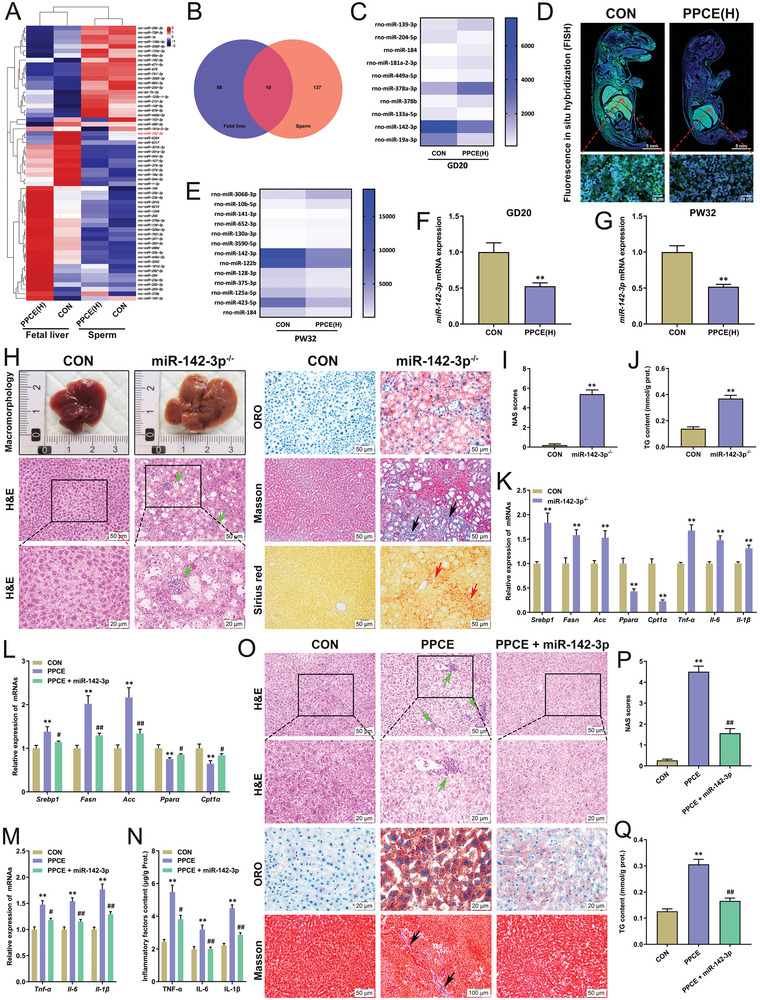
miR‐142‐3p is a critical target mediating PPCE‐induced hepatic lipid metabolism disorders and chronic inflammation in male offspring. A) Heatmap of gene expression in parental sperms and fetal livers; B) Venn diagram of miRNAs expression in parental sperms and fetal livers; C) Liver miRNAs expression at GD20; D) miR‐142‐3p intensity in fetal rat organs detected by FISH; E) Liver miRNAs expression at PW32; F,G) Liver miR‐142‐3p levels at GD20 and PW32 (*n* = 10); H) Liver appearance morphology, H&E, ORO, Masson, and Sirius red staining (200×, 400×); I,J) NAS score and liver TG content of miR‐142‐3p^−/−^ mice (*n* = 8); K) The mRNA expression of *Srebp1*, *Fasn*, *Acc*, *Pparα*, *Cpt1α*, *Tnf‐α, Il‐6*, and *Il‐1β* in liver of miR‐142‐3p^−/−^ mice (*n* = 8); L) The mRNA expression of *Srebp1*, *Fasn*, *Acc*, *Pparα*, and *Cpt1α* in liver at PW32 (*n* = 10); M,N) The content and mRNA expression of TNF‐*α*, IL‐6, IL‐1*β* in liver at PW32 (*n* = 10); O) Liver H&E, ORO, Masson, and Sirius red staining at PW32 (200×, 400×); P,Q) NAS score and liver TG content at PW32 (*n* = 10). Mean ± S.E.M. ^*^ *P <* 0.05, ^**^ *P <* 0.01 versus CON group; ^#^ *P <* 0.05, ^##^ *P <* 0.01 versus PPCE group (two‐tailed *t*‐test or one‐way ANOVA). Green arrow: inflammatory infiltration. Black and red arrows: deposition of collagen fibers.

Sperm miRNAs play a key role in the inheritance of acquired traits as a carrier of paternal genetic information and one of the important markers of epigenetic modifications.^[^ ^37^ ^]^ Therefore, we first examined the changes in liver miRNA expression profiles in paternal sperm and male offspring rats before and after birth to identify co‐differentially expressed miRNAs as potential toxicity targets. Sequencing analysis showed that compared with the CON group, the expression of multiple miRNAs was changed in paternal sperm and fetal liver of offspring in the PPCE group (**Figure** **3**A), with ten kinds of miRNAs being co‐differentially expressed (Figure 3B). Notably, miR‐142‐3p showed the most significant changes (Figure 3C). In situ hybridization experiments showed that the fluorescence‐labelled expression of miR‐142‐3p was considerably higher in the fetal liver than that in other organs, indicating its strong organ specificity (Figure 3D). Further, sequencing analysis suggested that the differential change of miR‐142‐3p was most significant in the liver of the PPCE group at PW32 (Figure 3E). RT‐qPCR results showed a significant reduction in hepatic miR‐142‐3p expression in the PPCE group at GD20 and PW32 (Figure 3F,G). These results suggest that PPCE reduces miR‐142‐3p expression in paternal sperm, which is then transmitted to the liver of male offspring, resulting in decreased hepatic miR‐142‐3p expression before and after birth.

Next, to confirm the regulatory effects of miR‐142‐3p on hepatic lipid metabolism and inflammatory responses, a liver‐specific miR‐142‐3p silencing (miR‐142‐3p^−/−^) mouse model was established using hydrodynamic injection and observed after 8 W. The liver of the miR‐142‐3p^−/−^ group appeared pale yellow and showed severe histological macro‐vesicular‐like steatosis with significant lipid accumulation, inflammatory infiltration (green arrows), and collagen fiber deposition (black and red arrows) (Figure 3H). Both liver NAS and triglyceride content were significantly elevated (Figure 3I,J). RT‐qPCR results showed that the expressions of hepatic lipogenesis genes (*Srebp1*, *Fasn*, and *Acc*) and pro‐inflammatory factor‐related genes (*Tnf‐α*, *Il‐6*, and *Il‐1β*) were significantly increased in the miR‐142‐3p^−/−^ group (Figure 3K), while the expressions of *Pparα* and *Cpt1α* were significantly decreased (Figure 3K). Additionally, miR‐142‐3p^−/−^ was found to promote hepatic STING‐related inflammatory signaling activation (Figure S3, Supporting Information). These results reveal that miR‐142‐3p^−/−^ could induce hepatic lipid metabolism dysfunction and inflammatory activation in mice, ultimately leading to the occurrence of NASH.

To further validate that miR‐142‐3p low expression programming mediated alterations in hepatic lipid metabolism in PPCE male offspring, a reverse intervention was performed by hydrodynamic injection of liver‐targeted AAV8‐miR‐142‐3p (miR‐142‐3p) overexpressing adeno‐associated virus at PW8. The results showed that the liver lipogenesis genes (*Srebp1*, *Fasn*, and *Acc*) expression was significantly decreased, *β*‐oxidation genes (*Pparα* and *Cpt1α*) expression was remarkably increased by miR‐142‐3p overexpression in PPCE offspring at PW32 (Figure 3L), and the content and mRNA expression of liver pro‐inflammatory factors (TNF‐*α*, IL‐6, and IL‐1*β*) were significantly decreased (Figure 3M,N). Histological results demonstrated that no obvious steatosis, lipid accumulation, or inflammatory infiltration were observed in the liver of miR‐142‐3p overexpressed PPCE offspring (Figure 3O), and both liver NAS and triglyceride content were significantly reduced (Figure 3P,Q). These results indicate that overexpression of miR‐142‐3p can reverse hepatic lipid metabolism dysfunction and inflammatory response in PPCE male offspring rats and inhibit adult NASH occurrence.

### 2.4 miR‐142‐3p Targets ACSL4 to Regulate Hepatic Lipid Metabolism and Inflammatory Responses in PPCE Male Offspring Mice

**Figure 4:**
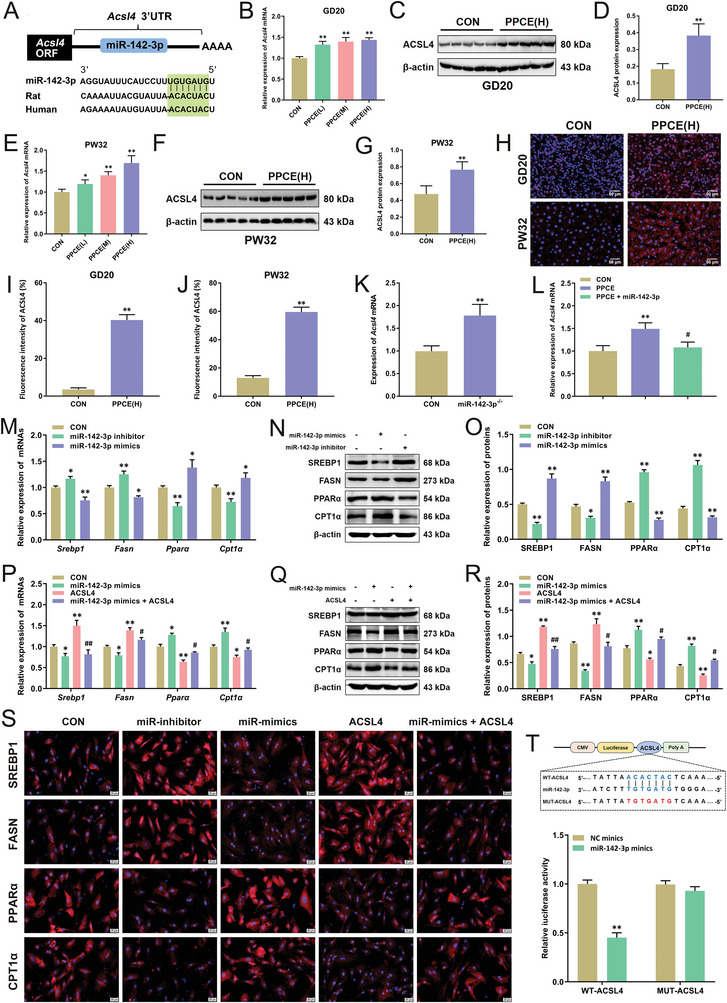
miR‐142‐3p targets ACSL4 to regulate hepatic lipid metabolism and inflammatory responses in PPCE male offspring mice. A) miR‐142‐3p and ACSL4 interactions; B) The mRNA expression of *Acsl4* at GD20 (*n* = 12); C,D) Representative immunoblots and quantitative analysis of ACSL4 at GD20 (*n* = 5); E) The mRNA expression of *Acsl4* at PW32 (*n* = 12); F,G) Representative immunoblots and quantitative analysis of ACSL4 at PW32 (*n* = 5); H–J) Representative immunofluorescent images and quantitative analysis of ACSL4 at GD20 and PW32 (*n* = 5); K) The expression of *Acsl4* mRNA in miR‐142‐3p^−/−^ mice (*n* = 8); L) The mRNA expression of *Acsl4* at PW32 (*n* = 10); M) The mRNA expression of *Srebp1*, *Fasn*, *Pparα*, and *Cpt1α* in BMSCs liver‐like differentiated cells after miR‐142‐3p inhibitor or miR‐142‐3p minics transfection (*n* = 6); N,O) Representative immunoblots and quantitative analysis of SREBP1, FASN, PPAR*α*, and CPT1*α* protein in BMSCs liver‐like differentiated cells after miR‐142‐3p inhibitor or miR‐142‐3p mimics transfection (*n* = 5); P) The mRNA expression of *Srebp1*, *Fasn*, *Pparα*, and *Cpt1α* in BMSCs liver‐like differentiated cells after miR‐142‐3p mimics and/or ACSL4 transfection (*n* = 6); Q,R) Representative immunoblots and quantitative analysis of SREBP1, FASN, PPAR*α*, and CPT1*α* protein in BMSCs liver‐like differentiated cells after miR‐142‐3p mimics and/or ACSL4 transfection; S) Representative immunofluorescence images of SREBP1, FASN, PPAR*α*, CPT1*α* in BMSCs liver‐like differentiated cells (*n* = 5); T) Dual luciferase reporter gene validation of miR‐142‐3p target gene (*n* = 6). Mean ± S.E.M. ^*^ *P <* 0.05, ^**^ *P <* 0.01 versus CON group; ^#^ *P <* 0.05, ^##^ *P <* 0.01 versus PPCE group (two‐tailed *t*‐test or one‐way ANOVA).

It is recognized that miRNAs can specifically bind to target gene mRNAs, regulating gene expression and playing important roles in processes such as embryonic development, cellular metabolism, and signal transduction.^[^ ^38^ ^]^ To elucidate the molecular mechanism by which miR‐142‐3p low expression mediates lipid metabolism disorder in the liver of PPCE male offspring rats, target genes of miR‐142‐3p were predicted using target databases (*TargetScan*, *miRDB*, and *miRcode*). The results identified long‐chain acyl‐CoA synthetase 4 (ACSL4) as a gene interacting with miR‐142‐3p (**Figure** **4**A). Subsequent examination of hepatic ACSL4 expression before and after birth showed that compared with the CON group, both mRNA and protein expression of liver ACSL4 were significantly increased in the PPCE group at GD20 and PW32 (Figure 4B–E). Immunofluorescence staining also demonstrated a significant increase in hepatic ACSL4 protein expression in the PPCE group before and after birth (Figure 4H–J). Furthermore, the expression of *Acsl4* mRNA was significantly elevated in the liver of miR‐142‐3p^−/−^ mice (Figure 4K), while miR‐142‐3p overexpression markedly reduced the expression of *Acsl4* mRNA in the liver of PPCE male offspring (Figure 4L). These results suggested that miR‐142‐3p might mediate changes in hepatic lipid metabolism in PPCE male offspring by targeting ACSL4.

Further, we confirmed the molecular mechanism by which miR‐142‐3p low expression mediated lipid metabolism dysfunction in the liver of PPCE male offspring using BMSCs hepatoid differentiated cells. First, transfection with miR‐142‐3p inhibitor and miR‐142‐3p mimics was used to confirm the regulatory effect of miR‐142‐3p on lipid metabolism function in hepatocytes. Compared with the CON group, miR‐142‐3p inhibitor significantly increased the expression of lipogenesis genes (SREBP1 and FASN) and decreased the expression of *β*‐oxidation‐related genes (PPAR*α* and CPT1*α*) (Figure 4M–O), while miR‐142‐3p mimics could reverse these changes. Meanwhile, miR‐142‐3p inhibitor significantly elevated the mRNA level and protein expression of ACSL4 in BMSCs liver‐like differentiated cells, whereas miR‐142‐3p mimics significantly decreased the mRNA level and protein expression of ACSL4 (Figure S4, Supporting Information). It was further confirmed that miR‐142‐3p regulated lipid metabolism by targeting ACSL4. Overexpression of ACSL4 significantly increased the expression of the aforementioned lipid synthesis genes and decreased the expression of *β*‐oxidation‐related genes (Figure 4P–R), and miR‐142‐3p mimics could reverse the changes caused by ACSL4 overexpression (Figure 4P–R). Similar results were observed by immunofluorescence analysis (Figure 4S). The regulatory effect of miR‐142‐3p by targeting ACSL4 on lipid metabolism was also observed in a rat primary hepatocyte model (data not shown). Finally, the interaction between miR‐142‐3p and ACSL4 was confirmed using a dual luciferase reporter assay (Figure 4T). These results indicate that miR‐142‐3p regulates hepatic lipid metabolism by targeting ACSL4.

### 2.5 High Glucocorticoid‐Induced Sperm miR‐142‐3p Methylation Reprogramming Mediates Hepatic Lipid Metabolism Dysfunction in PPCE Male Offspring

**Figure 5:**
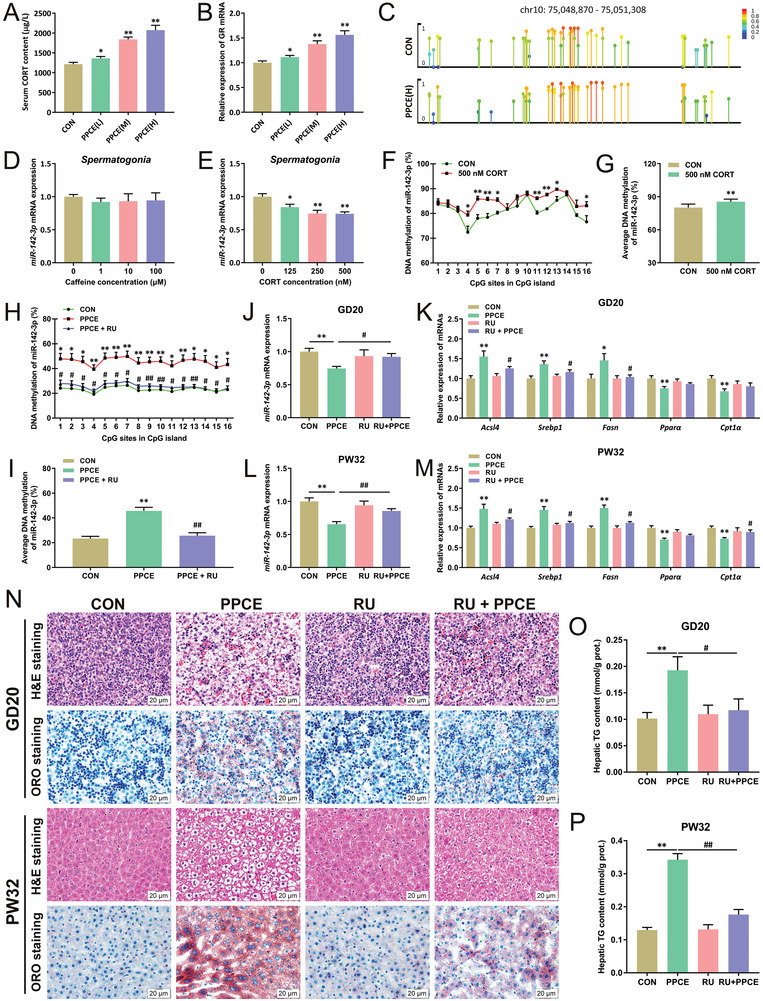
High glucocorticoid‐induced sperm miR‐142‐3p methylation reprogramming mediates hepatic lipid metabolism dysfunction in PPCE male offspring. A) Paternal serum CORT content (*n* = 10); B) The mRNA expression of GR in paternal testis (*n* = 10); C) Methylation levels of miR‐142‐3p in paternal sperms; D,E) The mRNA expression of miR‐142‐3p in spermatogonia after caffeine or CORT treated (*n* = 6); F,G) Methylation levels of miR‐142‐3p in CORT treated spermatogonia (*n* = 6); H,I) Methylation levels of miR‐142‐3p in liver at GD20 (*n* = 3); J,K) The mRNA expression of miR‐142‐3p, *Acsl4*, *Srebp1*, *Fasn*, *Pparα*, *Cpt1α* in liver at GD20 (*n* = 10); L,M) The mRNA expression of miR‐142‐3p, *Acsl4*, *Srebp1*, *Fasn*, *Pparα*, *Cpt1α* in liver at PW32 (*n* = 10); N) Liver H&E and ORO staining at GD20 and PW32 (400×); O,P) Liver TG content in GD20 and PW32 (*n* = 10). Mean ± S.E.M. ^*^ *P <* 0.05, ^**^ *P <* 0.01 versus CON group; ^#^ *P <* 0.05 versus PPCE group (two‐tailed *t*‐test or one‐way ANOVA).

Studies have shown that sperm “imprinting” with paternal epigenetic information from adverse environmental exposures significantly influences the long‐term health of the offspring.^[^ ^39^ ^]^ miRNAs and their methylation modifications are key epigenetic marks in the reprogramming of germ cells, transmitting the “imprint” of the parent's adverse environmental experiences to the offspring.^[^ ^32^, ^40^ ^]^ It was found that paternal chronic stress could lead to epigenetic modifications in sperm via high glucocorticoids, thereby programming abnormal liver glucose metabolism in the offspring.^[^ ^32^ ^]^ This suggests that high glucocorticoids might be an important mechanism for the occurrence of parental metabolic diseases. Therefore, in this study, the serum corticosterone level, testicular GR expression level, and methylation level of sperm miR‐142‐3p promoter region were first detected in the parental male rats. Compared with the CON group, these levels were significantly increased in the PPCE group (**Figure** **5**A–C). Further, mouse spermatogonia was treated in vitro with varying concentrations of caffeine (1–100 µm) or corticosterone (125–500 nm) to ascertain the cause of reduced sperm miR‐142‐3p expression. The results showed that caffeine treatment had no obvious effect on miR‐142‐3p expression in spermatogonia (Figure 5D), while corticosterone treatment significantly decreased miR‐142‐3p expression levels in spermatogonia (Figure 5E) and significantly increased the methylation level of the miR‐142‐3p promoter region (Figure 5F,G). To determine whether a strong relationship between high glucocorticoid exposure and sperm miR‐142‐3p promoter region methylation also existed in humans, a set of human samples was collected for assays and correlation analyses. The results showed that serum corticosterone levels in fertile males were significantly and positively correlated with sperm miR‐142‐3p promoter region methylation levels (Figure S5, Supporting Information), and these methylation levels were significantly and positively correlated with sperm viability concentration/aberration rate (Figure S5, Supporting Information). It is suggested that parental corticosterone (but not caffeine) programs the hypermethylation of miR‐142‐3p in PPCE paternal sperm.

To corroborate the involvement of parental high glucocorticoid exposure and sperm abnormalities in PPCE‐induced NAFLD susceptibility in male offspring, a series of intervention experiments were conducted. First, GR antagonist RU486 was used to synchronize interventions in PPCE rats to confirm the programming mechanism of paternal high‐glucocorticoid‐mediated alterations in hepatic lipid metabolism. Compared with the CON group, the methylation rate of the miR‐142 promoter region in the liver was increased and its expression was significantly decreased in the PPCE group at GD20 and PW32 (Figure 5H–K). Furthermore, expressions of hepatic lipogenesis function genes were significantly increased while those of *β*‐oxidation function genes were decreased (Figure 5L,M), accompanied by significantly increased hepatic lipid accumulation and triglyceride content (Figure 5N–P). Nonetheless, RU486 intervention significantly mitigated these changes in male offspring before and after birth (Figure 5H–P). It is indicated that parental high glucocorticoid exposure mediates alterations in hepatic lipid metabolism function in PPCE male offspring.

**Figure 6:**
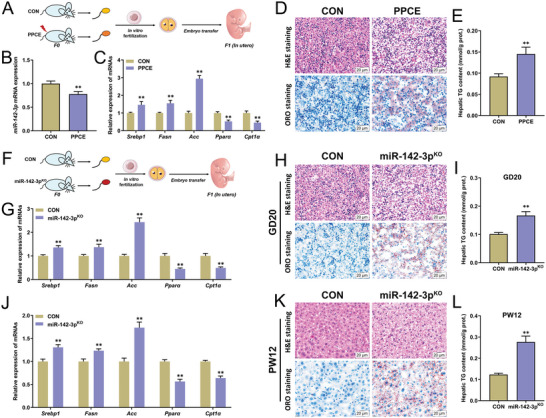
In vitro fertilization confirms that low expression of miR‐142‐3p mediates liver lipid metabolic changes in PPCE male offspring. A) Study design of in vitro fertilization in sperm of PPCE mice; B) The mRNA expression of miR‐142‐3p in GD19 offspring of PPCE mice; C) The mRNA expression of *Srebp1*, *Fasn*, *Acc*, *Pparα*, *Cpt1α* in GD19 offspring of PPCE mice; D) H&E and ORO staining in GD19 offspring of PPCE mice (400×); E) Liver TG content in GD19 offspring of PPCE mice; F) Study design of in vitro fertilization in sperm of miR‐142‐3p^KO^ mice; G) The mRNA expression of *Srebp1*, *Fasn*, *Acc*, *Pparα*, *Cpt1α* in GD19 offspring of miR‐142‐3p^KO^ mice, H) H&E and ORO staining in GD19 offspring of miR‐142‐3p^KO^ mice (400×); I) liver TG content in GD19 offspring of miR‐142‐3p^KO^ mice; J) The mRNA expression of *Srebp1*, *Fasn*, *Acc*, *Pparα*, *Cpt1α* in PW12 offspring of miR‐142‐3p^KO^ mice; K) H&E and ORO staining in PW12 offspring of miR‐142‐3p^KO^ mice (400×); L) Liver TG content in PW12 offspring of miR‐142‐3p^KO^ mice. Mean ± S.E.M., *n* = 6, ^*^ *P <* 0.05, ^**^ *P <* 0.01 versus CON group (two‐tailed *t*‐test).

Next, the effect of fertilization between PPCE mouse sperm and normal oocytes on changes in liver lipid metabolism function in male offspring was explored using in vitro fertilization technique (**Figure** **6**A). Compared with the CON group, the expression of miR‐142‐3p was decreased and the expressions of lipogenesis genes (*Srebp1*, *Fasn*, and *Acc*) were significantly increased while the expressions of *β*‐oxidation genes (*Pparα* and *Cpt1α*) were significantly decreased in the fetal liver of the offspring in the PPCE group (Figure 6B,C). Histological results showed that numerous fat‐like vacuoles, lipid accumulation, and significantly elevated liver triglyceride content were observed in the fetal liver of the PPCE group (Figure 6D,E). Further, to confirm that low expression of sperm miR‐142‐3p could mediate changes in liver lipid metabolism function in male offspring, the effect of miR‐142‐3p^KO^ sperm on changes in liver lipid metabolism function in male offspring after fertilization with normal oocytes was investigated (Figure 6F). The results showed that the expressions of lipogenesis genes were significantly increased in the fetal liver of the miR‐142‐3p^KO^ group (Figure 6G), while the mRNA expressions of fatty acid *β*‐oxidation function genes were decreased (Figure 6G), and the fetal liver showed obvious steatosis and significantly increased triglyceride content (Figure 6H,I). At PW12, the liver lipogenesis was enhanced, *β*‐oxidation was diminished in the miR‐142‐3p^KO^ group, and extensive vacuolar‐like steatosis along with excessive lipid accumulation was observed in the liver and NAFLD occurred (Figure 6J–L). It was suggested that low expression of sperm miR‐142‐3p could mediate hepatic lipid metabolism dysfunction and the occurrence of NAFLD in male offspring.

### 2.6 PPCE Induces NASH Occurrence in Male Offspring with Transgenerational Inheritance

**Figure 7:**
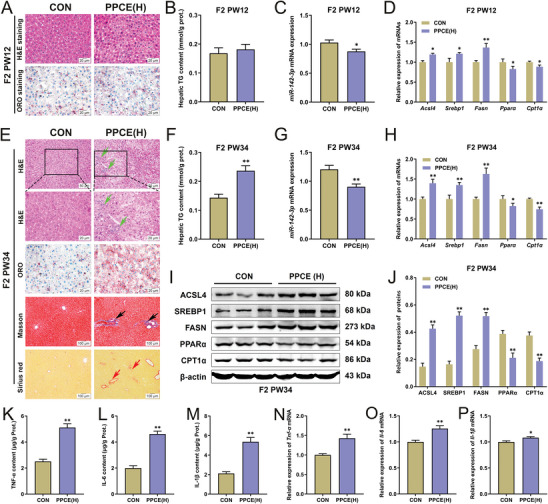
PPCE‐induced NASH occurrence in male offspring with transgenerational inheritance. A) H&E and ORO staining at PW12 (400×); B) Liver TG content at PW12 (*n* = 10); C) The mRNA expression of miR‐142‐3p at PW12 (*n* = 10); D) The mRNA expression of *Acsl4*, *Srebp1*, *Fasn*, *Pparα*, and *Cpt1α* at PW12 (*n* = 10); E) H&E, ORO, Masson, and sirius red staining at PW34 (400×); F) Liver TG content at PW34 (*n* = 10); G) The mRNA expression of miR‐142‐3p at PW34 (*n* = 10); H) The mRNA expression of *Acsl4*, *Srebp1*, *Fasn*, *Pparα*, and *Cpt1α* at PW34 (*n* = 10); I,J) Representative immunoblots and quantitative analysis of ACSL4, SREBP1, FASN, PPAR*α*, and CPT1*α* protein at PW34 (*n* = 5); K–M) The contents of TNF‐*α*, IL‐6 and IL‐1*β* at PW34 (*n* = 10); (N‐P) The mRNA expression of *Tnf‐α*, *Il‐6* and *Il‐1β* at PW34 (*n* = 10). Mean ± S.E.M. ^*^ *P <* 0.05, ^**^ *P <* 0.01 versus CON group (two‐tailed *t*‐test).

To clarify whether PPCE has a transgenerational genetic effect on adult NASH in male offspring, changes in liver histology, lipid metabolism function, and inflammatory response in the F2 generation of the offspring were observed. Compared with the CON group, no significant changes in liver pathology and triglyceride content were noted in the F2 generation of PPCE offspring at PW12 (**Figure** **7**A,B), but miR‐142‐3p expression was significantly decreased (Figure 7C), *Acsl4* and lipogenesis genes (*Srebp1* and *Fasn*) expressions were significantly increased while *β*‐oxidation genes (*Pparα* and *Cpt1α*) expressions were decreased (Figure 7D). However, at PW34, a large number of hepatocytes in the liver of F2 generation in PPCE offspring showed steatosis with lipid accumulation, inflammatory infiltration (green arrows), and collagen fibril deposition (black and red arrows) (Figure 7E), and liver triglyceride content was significantly elevated (Figure 7F). RT‐qPCR and Western blot results showed that at PW34, liver miR‐142‐3p expression was significantly decreased in the F2 generation of PPCE offspring (Figure 7G), the expression of ACSL4 and lipogenesis genes were significantly increased, while the expression of *β*‐oxidation genes were decreased (Figure 7H–J). Additionally, liver pro‐inflammatory factor content (Figure 7K–M) and mRNA expression (Figure 7N–P) were significantly increased in the F2 generation of PPCE offspring at PW34. These findings suggested that PPCE could cause hepatic lipid metabolism dysfunction, inflammatory response activation, and adult NASH occurrence in the F2 generation of male offspring, thereby demonstrating that PPCE‐induced adult NASH occurrence in male offspring included transgenerational inheritance.

## 3 Discussion

Developmental toxicity of caffeine, in pregnant women, children, and adolescents, has been confirmed.^[^ ^41^, ^42^, ^43^, ^44^ ^]^ According to the European Food Safety Authority's report, daily caffeine intake should not exceed 400 mg in healthy adults.^[^ ^44^ ^]^ However, excessive consumption remains prevalent, especially in Europe and the United States.^[^ ^22^ ^]^ Although our previous studies have revealed the impacts of maternal caffeine exposure during pregnancy on the long‐term health of the offspring,^[^ ^18^, ^19^, ^20^ ^]^ the effect of xenobiotics‐induced chronic stress on the father and the progeny, such as PPCE, should not be ignored. In this study, we established a PPCE rat model using caffeine doses of 15, 30, and 60 mg kg^−1^/d, equivalent to ≈0.5, 1, and 2‐fold of the recommended safe dose for humans, converted according to body surface area. Our findings showed that PPCE caused NASH in adult male offspring rats with transgenerational inheritance, attributed to hypermethylation of sperm miR‐142‐3p programmed by paternal high glucocorticoid, confirming that miR‐142‐3p is a key regulator of lipid metabolism and a potential interventional target for paternal stress‐driven NASH.

As important epigenetic modification markers, miRNAs are responsible for transferring the impacts of adverse factors in father to offspring, causing long‐term health problems.^[^ ^30^, ^32^ ^]^ In this study, miR‐142‐3p was identified as the specific miRNA mediating the acquired hepatic phenotype of male F1 generation from PPCE‐F0 rat sperm. PPCE caused a significant reduction in hepatic miR‐142‐3p expression in male offspring, and liver‐specific silencing of miR‐142‐3p could induce NASH in mice. These findings suggested that miR‐142‐3p deficiency mediates PPCE‐induced NASH occurrence in male offspring. ACSLs are key enzymes for acyl‐CoA synthesis and are essential in regulating fatty acid metabolism.^[^ ^45^ ^]^ As a subtype of the ACSLs family, ACSL4 is most tightly involved in fatty acid metabolism by promoting the fatty acid *de novo* synthesis process in hepatocytes through activation of transcription factor SREBP1 and inhibiting fatty acid *β*‐oxidation through suppression of PPAR*α* transcription, thus playing an important role in the development of liver metabolic diseases.^[^ ^46^, ^47^ ^]^ ACSL4 is a predicted target of miR‐142‐3p, and the relationship between ACSL4 and miR‐142‐3p was verified in BMSCs, accompanied by corresponding lipid metabolism changes. In addition, up‐regulation of ACSL4 paralleled to low level of miR‐142‐3p in the liver of PPCE male offspring. Collectively, these results suggest that low expression of miR‐142‐3p contributes to hepatic lipid metabolism disorder through the upregulation of ACSL4.

It has been demonstrated that excessive lipid accumulation in the liver results in lipotoxicity, triggering mitochondrial oxidative stress and inflammatory responses to accelerate the progression of NAFLD.^[^ ^48^ ^]^ STING, a critical component of innate immunity located on the endoplasmic reticulum, regulates inflammatory responses by activating transcription factors such as NF‐κB and IRF3.^[^ ^49^ ^]^ Our findings reveal that PPCE increases oxidative stress and activates STING‐mediated inflammatory signaling in liver macrophages of male offspring, resulting in increased production of pro‐inflammatory factors in the liver, including TNF‐*α*, IL‐6, and IL‐1*β*. Moreover, the absence of miR‐142‐3p (miR‐142‐3p^−/−^) enhances STING‐mediated inflammatory response activation in the liver. This, in turn, mediates the occurrence of NASH in male offspring.

Clinical studies reported that miR‐142‐3p expression was reduced in peripheral blood mononuclear cells (PBMCs) of children with metabolic‐associated fatty liver disease (MAFLD), and was potentially correlated with serum triglyceride levels,^[^ ^50^ ^]^ suggesting a close association between miR‐142‐3p and the occurrence of MAFLD from a clinical insight. Despite the growing evidence of NAFLD's paternal origin, the programming mechanisms remain unclear, leading to difficulties in early prevention and treatment. Related research has shown that improving paternal pre‐pregnancy lifestyle, such as proper exercise and antioxidant supplementation, has beneficial effects on preventing paternal NAFLD.^[^ ^51^, ^52^ ^]^ However, due to individual differences and a lack of specificity, these measures cannot be considered effective preventive or treatment strategies for paternal‐derived NAFLD/NASH. In the present study, we found that miR‐142‐3p deficiency precipitates PPCE‐induced NASH in male offspring while overexpressing miR‐142‐3p reversed the NASH occurrence in male offspring. Additionally, in vitro fertilization experiments further confirmed that miR‐142‐3p low expression could program the alteration of hepatic lipid metabolism in PPCE male offspring. Intriguingly, we also observed low expression of hepatic miR‐142‐3p and lipid metabolism dysfunction in rat male offspring whose father was exposed to mixed factors including nicotine, ethanol, and caffeine (Figure S6, Supporting Information). It was suggested that miR‐142‐3p could serve as a potential target for preventing and treating paternal‐stress‐driven NAFLD in offspring. This finding paves a new way to develop nucleic acid drugs for NAFLD treatment.

Glucocorticoids are known to be the most important regulatory hormone in systematic stress response and play a crucial role in germ cell formation, fetal maturation, and postnatal fate.^[^ ^25^ ^]^ Studies have shown that paternal adverse environmental exposure can provoke HPA axis activation, leading to a chronic stress state of the body^[^ ^53^, ^54^ ^]^ and affecting sperm epigenetic modifications through elevated serum glucocorticoid levels, which result in offspring dysplasia.^[^ ^32^, ^55^ ^]^ It is suggested that high glucocorticoid‐reprogrammed sperm epigenetic modifications may be an important mechanism mediating the transmission of paternally acquired traits to the offspring. DNA methylation, one of the most important forms of epigenetic inheritance in eukaryotes, facilitates the inheritance of paternally acquired traits.^[^ ^56^ ^]^ Clinical evidence suggests that poor paternal dietary habits can alter DNA methylation during sperm reprogramming, thereby increasing the risk of metabolic disease in the offspring after birth.^[^ ^57^, ^58^ ^]^ In this study, we observed significant elevations in serum corticosterone and the methylation level of the miR‐142‐3p promoter region in sperm and offspring liver. Moreover, it was corticosterone, but not caffeine, that caused the elevated methylation level of the miR‐142‐3p promoter region. To clarify the glucocorticoid programming mechanism of PPCE‐induced hepatic lipid metabolism disorder and NASH occurrence, we used the GR antagonist (RU486) to intervene in PPCE rats and found that RU486 reduced the methylation level of the miR‐142‐3p promoter region in the sperm of PPCE‐F0 generation and liver of male F1 generation, and reversed the occurrence of NASH. Interestingly, we found a significant positive correlation between serum corticosterone levels and sperm miR‐142‐3p promoter region methylation in fertile men, as well as between sperm miR‐142‐3p promoter region methylation and sperm concentration/aberration rate in adult men (Figure S4, Supporting Information). It is suggested that high glucocorticoid‐programmed sperm miR‐142‐3p hypermethylation resulting from parental chronic stress mediates the occurrence of PPCE‐induced NASH in male offspring, where hypermethylation of sperm miR‐142‐3p promoter region is expected to be an early warning marker for clinical susceptibility to paternal‐derived NASH.

Transgenerational inheritance involves the transmission of epigenetic information through germ cells to subsequent generations, having profound implications for biological evolution and disease occurrence.^[^ ^59^ ^]^ Previous studies have shown that paternal pre‐pregnant exposure to adverse environmental factors had impacts on the health of offspring and subsequent generations.^[^ ^60^ ^]^ In the present study, we found that PPCE caused hepatic lipid metabolism dysfunction, steatosis, inflammatory infiltration, and collagen fibril deposition in the F2 male generation, which was similar to the F1 generation, suggesting that PPCE‐induced adult NASH occurrence in male offspring with transgenerational inheritance. Interestingly, the abnormal liver pathological alterations in male offspring of the PPCE‐F2 generation were significantly reduced compared to the PPCE‐F1 generation. We hypothesized that, although the changes in liver pathology in the F2 generation are likely to stem from the intergenerational transmission of PPCE‐induced sperm reprogramming in the F0 generation and programming effect in the F2 generation due to low glucocorticoid exposure in the F1 generation,^[^ ^19^ ^]^ further exploration into the mechanisms underlying these histopathological changes in the liver of the F2 generation are needed in the future.

Related studies have shown that developmental diseases often exhibit significant gender differences, which might be related to variations in maternal compensatory regulation on the fetal development across genders during intrauterine development of the embryo.^[^ ^61^ ^]^ In this study, we found that PPCE could lead to the onset of adult NAFLD in female offspring, but histological alterations were less typical than that of males, no significant inflammatory infiltration and collagen fibril deposition were observed in the liver and hepatic miR‐142‐3p expression levels were significantly reduced in PPCE male offspring before and after birth. Conversely, liver miR‐142‐3p expression in PPCE female offspring showed no significant changes (Figure S1, Supporting Information). In conclusion, PPCE‐induced NASH in adult offspring demonstrates gender‐specific differences, with female offspring displaying atypical disease characteristics. Several reproductive‐related hormones are strongly associated with gender differences in intrauterine fetal growth and development. In particular, estrogen and the estrogen receptor signaling pathway have been proven to play an important role in achieving gender differences in fetal growth and development.^[^ ^62^, ^63^ ^]^ Recent research has revealed that gender differences in altered glycolipid metabolism in offspring due to paternal adverse environmental exposures are tightly connected to the hepatic estrogen signaling pathway, whereby enhanced estrogen and estrogen receptor signaling protected female offspring against adverse environment.^[^ ^64^ ^]^ This protective effect might originate from intrauterine maternal compensatory protection of fetal growth and development.^[^ ^63^ ^]^ Interestingly, we also found that the PPCE‐induced abnormal development of multiple organs (including liver, adrenal glands, bone, and cartilage) in female offspring was significantly less severe than that in male offspring, which may be attributed to the active, compensatory regulation of intrauterine offspring development by the mother. Future studies will explore the gender‐specific functional abnormalities of multiple organs in the offspring due to PPCE and the pathogenesis.

**Figure 8:**
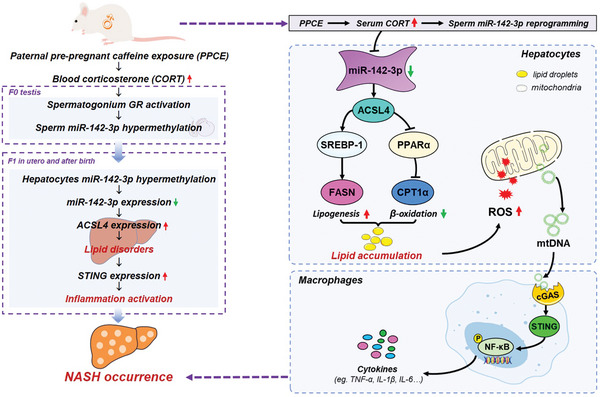
Schematic diagram of miR‐142‐3p low expression‐mediated hepatic lipid dysregulation in male offspring of paternal pre‐pregnant caffeine exposure.

In summary, this study provides the pioneering demonstration of the link between PPCE and disrupted hepatic lipid metabolism in male offspring, leading to the development of NASH in adulthood. The underlying mechanism involves elevated glucocorticoid levels from PPCE, which induce hypermethylation in the sperm miR‐142‐3p promoter region. This epigenetic modification persists in the livers of male offspring, contributing to diminished miR‐142‐3p expression, subsequent dysfunction in hepatic lipid metabolism, and the activation of inflammatory pathways, ultimately leading to adult‐onset NASH (**Figure** **8**). Importantly, hepatic overexpression of miR‐142‐3p effectively reversed the occurrence of NASH in PPCE‐exposed male offspring, providing a potential intervention strategy for high glucocorticoid‐mediated programming. This research elucidates both the developmental origins and programming mechanisms of paternal NASH and confirms a viable intervention target.

## 4 Experimental Section

### Reagents

Caffeine (purity ≥ 99.0%) and corticosterone were purchased from Sigma–Aldrich (St Louis, MO, USA). Mifepristone (RU486) was obtained from Hubei Gedian Humanwell Pharmaceutical Co. LTD (Ezhou, China). Rat‐miR‐142‐3p inhibitor oligo, rat‐miR‐142‐3p mimics oligo, rat‐ACSL4 plasmid, and mus‐miR‐142‐3p siRNA were obtained from GenePharma (Shanghai, China). AAV8 adenovirus delivery‐miR‐142‐3p was purchased from Tsingke Biotechnology (Beijing, China). miScript SYBR Green PCR Kit and SYBR Green qPCR Master Mix were purchased from Vazyme biotech (Nanjing, China).

### Animal Treatment

Specific pathogen‐free (SPF) Wistar rats were purchased from the Experimental Center of the Hubei Medical Scientific Academy and accommodated to standard nutritional conditions with a 12 h light/dark cycle. All animal experiments were performed according to protocols approved by the Institutional Animal Care and Use Committee at Wuhan University (WHEF‐2022‐0286).

Male Wistar rats (F0) were randomly ranged into the control and paternal pre‐pregnant caffeine exposure (PPCE) groups (*n* = 12). Animals in the PPCE group were administrated with different concentrations of caffeine (15, 30, and 60 mg kg^−1^/d) for 8 consecutive weeks, and the control group rats were treated with the same volume of normal saline. After caffeine administration, male rats were mated with normal females in a joint cage. The day when sperm appeared in the vaginal smear was designated as gestational day (GD) 0. Half of the pregnant rats were euthanized on GD20 with 3% isoflurane, liver was collected for further analysis. The rest pregnant rats were kept to obtain the F1 generation. One day after birth, the number of pups was standardized to 12 pups per litter (six males and six females). The offspring were fed with normal diet after weaning at postnatal week 3 (PW3) and sacrificed under isoflurane anesthesia at PW12 and PW32, respectively (*n* = 12, randomly chose one from each litter). Liver tissues were collected for subsequent analysis. A subset of F1 male rats was randomly selected on PW8, and divided into vector and AAV8‐miR‐142‐3p group (*n =* 20). AAV8‐miR‐142‐3p and vectors were injected respectively into rats by hydrodynamic injection via tail vein. These rats were then sacrificed at PW12 (*n =* 10) and PW32 (*n =* 10).

To further investigate the underlying mechanism, F0 male rats were randomly divided into the control (normal saline) group, PPCE (60 mg kg^−1^/d caffeine) group, RU486 (1 mg kg^−1^/d) group,^[^ ^32^, ^65^ ^]^ and RU486 (1 mg kg^−1^/d) + PPCE (60 mg kg^−1^/d caffeine) group (*n* = 12). Rats in each group were administrated with corresponding reagents daily for continuous 8 weeks and then mated with normal female rats at the end of the administration. Half of the pregnant rats were killed at GD20, and the rest pregnant rats delivered the F1 generation naturally. The F1 rats were sacrificed at PW12 and PW32 (*n* = 12). Moreover, another part of F1 male rats was mated with normal female rats at PW12 to produce the F2 generation, and F2 rats were sacrificed at PW12 and PW34 (*n =* 12), respectively.

### Human Plasma and Sperm Samples

Human plasma and sperm samples were collected from 28 male patients who underwent assisted reproductive tests in the Reproductive Medicine Center of Zhongnan Hospital, Wuhan University. The plasma cortisol concentration, sperm general characteristics (sperm concentration, sperm number, sperm malformation rate, etc.), and sperm miR‐142‐3p promoter region methylation rate were detected, and the correlation between the above results was analyzed. The study was approved by the Institutional Ethics Committee of the Zhongnan Hospital of Wuhan University (No. 2020188). Informed consent was obtained from the patients before the collection of plasma and sperm samples. All the research was performed by government policies and the Helsinki Declaration.

### Hydrodynamic Injection

The mus‐miR‐142‐3p inhibitor lentivirus plasmid (miR‐142‐3p^−/−^) was synthesized by Genepharma (Shanghai, China), and its sequence is shown in Table S1 (Supporting Information). Male FVB/N mice (20–22 g) were obtained from Charles River Laboratories (Beijing, China), and randomly divided into control and miR‐142‐3p^−/−^ groups (*n* = 8). The hydrodynamic injection was performed to conduct a hepatic‐specific miR‐142‐3p^−/−^ mouse model as previously described.^[^ ^66^ ^]^ In detail, 20 µg miR‐142‐3p^−/−^ plasmids were diluted in 2 mL normal saline, then plasmids solution with a volume of 10% of the animal's body weight was transiently injected into the tail‐vein of mice in miR‐142‐3p^−/−^ group within 6–7 s. Mice in the control group were also injected with the same volume of the vehicle. After 8 weeks, all mice were euthanized with 3% isoflurane to collect serum and liver samples for the following analysis.

### In Vitro Fertilization and Embryo Culture

Male C57BL/6 mice were administrated with a gavage of caffeine (120 mg kg^−1^/d) for continuous 8 weeks to establish a PPCE mice model (*n* = 12). The control group was also administered the same amount of saline. Sperm were isolated from the epididymis after the administration and were diluted to a concentration of 2 × 10^6^/mL in the tubal fluid medium, followed by further investigation after 15 min capacitation. Female C57BL/6 mice (*n* = 24) were injected with 5 IU equine chorionic gonadotropin and 5 IU human chorionic gonadotropin for superovulation, followed by collecting mature oocytes from the fallopian tube. In vitro fertilization was performed as previously described.^[^ ^67^ ^]^ Briefly, sperms of mice in the control or PPCE group and normal oocytes were transferred into the embryo medium for further culture and fertilization to get fertilized eggs. Fertilized zygotes were cultured in vitro for 48 h, then the surviving embryos were transferred into the oviduct of pseudopregnant recipient female mice. All pregnant mice were anesthetized using 3% isoflurane to strip fetal mice 20 days after embryo transfer and collect fetal blood and liver samples for further investigations.

Male miR‐142‐3p knockout (miR‐142‐3p^KO^) C57BL/6 mice (*n* = 6) were generated by Cyagen Biosciences (Suzhou, China) using CRISPR/Cas9 technology, and miR‐142‐3p^KO^ sperm were collected from the epididymis. Meanwhile, sperm and mature oocytes of normal male and female mice were also extracted. Normal sperm or miR‐142‐3p^KO^ sperm were fertilized with normal oocytes to generate fertilized oocytes, which were then transferred to pseudopregnant recipient female mice. Fetal blood and liver samples were harvested 20 days after embryo transfer as the above method. The rest of the pregnant mice were fed till natural birth. The offspring were raised to adulthood to determine the effect of miR‐142‐3p^KO^ on hepatic lipid metabolic function in male offspring.

### Cell Culture

Bone marrow mesenchymal stem cells (BMSCs) hepatoid differentiated cells were extracted as previously described.^[^ ^68^ ^]^ In brief, BMSCs were extracted from 3‐week‐old male rats and cultured using *α*‐MEM medium with 10% fetal bovine serum (FBS), 100 mg mL^−1^ streptomycin, and 100 U mL^−1^ penicillin. When the cells reached 80% confluence, they were incubated with hepatocyte differentiation medium (*α*‐MEM medium with 1% FBS, 100 mg mL^−1^ streptomycin, 100 U mL^−1^ penicillin, 20 ng mL^−1^ HGF, 2 ng mL^−1^ EGF, 0.1 µm dexamethasone, and 50 mg mL^−1^ ITS) for 14 days.^[^ ^69^ ^]^ 20 nm miR‐142‐3p inhibitor, 20 nm miR‐142‐3p mimics, or 2.5 µg ACSL4 plasmid were transfected into cells using lipofectamine 3000 (Thermo Fisher Scientific, Waltham, USA). The primer sequences of miR‐142‐3p mimics and miR‐142‐3p inhibitor were shown in Table S1 (Supporting Information). In detail, the plasmids and transfection reagent were incubated in DMEM for 20 min at room temperature to perform transfection into BMSCs hepatoid differentiated cells, and harvested for further assessment after 24 h. Mouse spermatogonial cell line GC‐1 cells (Procell Life Science &Technology, Wuhan, China) were also cultured under the above conditions. GC‐1 cells were treated with corticosterone (0, 125, 250, 500 nm) or caffeine (0, 1, 10, 100 µm) for 24 h, respectively. Cells were then collected to detect the mRNA expression level of miR‐142‐3p and the rate of promoter region methylation. All in vitro data were from triplicate independent experiments.

### DNA Methylation Analysis

The bisulfite pyrosequencing method was performed for DNA methylation analyses in sperm and liver samples (*n* = 3) as previously reported.^[^ ^70^ ^]^ DNA was extracted from F0 rat sperm and male F1 liver tissues using a Mammalian genomic DNA extraction kit (Beyotime, Shanghai, China). To measure the DNA permethylation status, the EZ DNA Methylation‐Gold Kit (ZYMO, CA, USA) was used for sample processing to convert unmethylated cytosine into uracil. After building the MethylTarget library, high‐throughput sequencing was employed with the Illumina HiSeq platform.

### Whole Transcriptome Sequencing

Sperm and liver samples of male rats were lysed with Trizol reagent for further analysis. The sequencing libraries were generated using the TruSeq Stranded Total RNA Library Prep Kit (Illumina, USA). Then, the library analysis was performed using the online platform of Majorbio Cloud Platform (www.majorbio.com) (Shanghai Majorbio Bio‐pharm Technology Co., Ltd). The analyses of mRNA and miRNA expression were conducted using the DESeq R package, and the transcripts with *P* < 0.05 and log2 fold change > 2 were selected as differentially expressed mRNAs and miRNAs. The enrichment analysis was plotted using the R software. All sequencing was analyzed on the Whole Transcriptome Cloud Platform of Shanghai Meggie Biotechnology (https://cloud.majorbio.com).

### Hematoxylin‐Eosin (H&E) Staining, oil red O (ORO) Staining, Masson Staining, Sirius Red Staining, and Transmission Electron Microscopy (TEM)

For histopathological analysis, fresh liver tissues were fixed within 10% paraformaldehyde and then embedded with paraffin to slice into liver sections. Liver slides were dewaxed with xylene and rehydrated with ethanol at decreasing concentration gradients to perform H&E staining, Masson staining, and Sirius red staining. For ORO staining, frozen liver slices were incubated within ORO solution, rinsed with 60% isopropanol, and the nucleus was redyed with hematoxylin. All slides were then mounted under coverslips for further observation under a microscope. The pathological grading and staging of NASH rats were scored according to the non‐alcoholic fatty liver diseases (NAFLD) activity score (NAS) system reported by *Kleiner* et al.^[^ ^71^ ^]^ The scoring system consisted of a semi‐quantitative assessment of three histological features demonstrated in liver H&E staining images (200×): steatosis (0‐3), lobular inflammation (0‐3), and hepatocellular ballooning (0‐2). NAS was the sum of steatosis, lobular inflammation and hepatocellular ballooning scores, in which NAS < 3 (non‐NASH), NAS > 4 (NASH), and NAS between 3 and 4 (possibly NASH).

For TEM analysis, fresh liver tissues from mice were fixed in 2.5% glutaraldehyde, post‐fixed in 1% OsO4, and dehydrated with gradient ethanol and acetone. The tissues were embedded in epoxy resin, cut into serial TEM ultrathin sections, and stained with uranyl acetate and lead citrate. The sections were photographed using a JEM 1400 JEOL instrument (Tokyo, Japan).

### Real‐Time Quantitative Polymerase Chain Reaction (RT‐qPCR)

Total RNA was extracted from liver homogenate and BMSCs hepatoid differentiated cells using TRIzol Reagent, and then converted into complementary DNA (cDNA). The RT‐qPCR assay was subsequently processed to determine the number of specific DNA sequences by FastStart Universal SYBR Green Master Mix (Roche, Basel, Switzerland) on a 384‐well ABI StepOnePlus cycler (Applied Biosystems, CA, USA). Glyceraldehyde‐3‐phosphate dehydrogenase (GAPDH) was taken as an internal reference for quantitative assay. NADH dehydrogenase subunit 1 (ND1) gene encoded by mtDNA and GAPDH encoded by nuclear DNA (nDNA) were determined by RT‐qPCR analysis as previously described.^[^ ^72^ ^]^ The mtDNA/nDNA ratio was used to assess the relative mtDNA copy number. All the identified primers are listed in Table S2 (Supporting Information).

### Western Blotting and Immunofluorescence

Western blotting was performed to determine protein expressions in liver tissues and BMSCs hepatoid differentiated cells. Briefly, the concentrations of extracted cytosol protein were determined using the BCA protein assay kits (Thermo Fisher Scientific, USA). Proteins were then separated with sodium dodecyl sulfate‐polyacrylamide gel electrophoresis (SDS‐PAGE), and transferred onto PVDF membranes. After blocking with 5% skim milk powder for at least 1 h, membranes were incubated with specific primary antibodies overnight, followed by incubating with corresponding second antibodies for 1 h. The bands were visualized by chemiluminescence using an electro‐chemiluminescent detection kit (Thermo Fisher Scientific). The involved primary antibodies are listed in Table S3 (Supporting Information).

For immunofluorescence, BMSCs hepatoid differentiated cell climbing slices and fixed liver slides were incubated with primary antibodies overnight at 4 °C. After that, slides were washed three times with PBS, incubated with DAPI and FITC‐conjugated second antibodies for 1.5 h at 37 °C, and observed under a fluorescence microscope. The involved primary antibodies are listed in Table S3 (Supporting Information).

### Fluorescence In Situ Hybridization (FISH)

The FISH assay was performed as previously described.^[^ ^73^ ^]^ The whole fetal rat was fixed within 10% paraformaldehyde and then embedded with paraffin to slice. Paraffin sections were dewaxed with xylene and rehydrated with ethanol at a decreasing concentration gradient to perform FISH. Fluorescent‐labeled probe for miR‐142‐3p (Genepharma, Shanghai, China) was applied during hybridization. FISH was performed using the RNAscope Multiplex Fluorescent Multiplex kit (Advanced Cell Diagnostics, California, USA) according to the manufacturer's protocol and observed under a fluorescence microscope.

### Dual‐Luciferase Reporter Gene Assay

After 50–70% of confluence, cells were co‐transfected with 2 µg pMiR‐report vector‐RAB22 A/SNHG3 3′UTR and miR‐142‐3p using Lipofectamine 2000 for 6 h, then lysed for luciferase activity determination using Dual‐luciferase Reporter Assay System. Luciferase reporter vectors were provided by GenePharma (Shanghai, China).

### Corticosterone Content Assay

Serum corticosterone content was determined by enzyme‐linked immunosorbent assay (ELISA) according to the manufacture's instruction (R&D Systems, Minneapolis, MN, USA). In detail, 50 µL of standard, control sample or serum sample to be tested was added to each well of the ELISA reaction plate for reaction, followed by determination of the absorbance on a microplate reader at 405 nm. The corticosterone content was then calculated and confirmed.

### Statistical Analysis

GraphPad Prism 8.0 software (GraphPad Software Inc., San Diego, CA, USA) was employed for statistical analysis and data visualization. All data were obtained from at least three independent experiments and expressed as Mean ± S.E.M. values. RT‐qPCR assay data were normalized to the control group. Statistical analysis was performed using the Two‐tailed Student's *t*‐test or one‐way ANOVA between two groups and among three or more groups, respectively. Sample size (*n*) for each statistical analysis were depicted in the figure legends. *P <* 0.05 represented statistical significance.

## Conflict of Interest

The authors declare no conflict of interest.

## Author Contributions

C.Z. and Y.G. contributed equally to this work. C.Z. performed conceptualization, data curation, and wrote the original draft. Y.G. performed methodology and investigation. Y.L. performed software and visualization. K.L. and W.H. performed formal analysis. H.W. performed project administration, funding acquisition, and wrote, reviewed and edited the final manuscript. All authors approved the manuscript for submission.

## Supporting information



Supporting Information

## Data Availability

The data that support the findings of this study are available from the corresponding author upon reasonable request.
