# Supplementary material for: Sperm miR‐142‐3p Reprogramming Mediates Paternal Pre‐Pregnancy Caffeine Exposure‐Induced Non‐Alcoholic Steatohepatitis in Male Offspring Rats
Source: Adv Sci (Weinh). 2024 Sep 18;11(42):2405592. doi: 10.1002/advs.202405592 (PMC11558112; doi:10.1002/advs.202405592)
Supplement: Supplementary file 1 — Supporting Information [file ADVS-11-2405592-s001.pdf]

ADVANCED SCIENCE
Open Access

Supporting Information

for *Adv. Sci.*, DOI 10.1002/advs.202405592

Sperm miR-142-3p Reprogramming Mediates Paternal Pre-Pregnancy Caffeine Exposure-Induced Non-Alcoholic Steatohepatitis in Male Offspring Rats

*Cong Zhang, Yu Guo, Yi Liu, Kexin Liu, Wen Hu and Hui Wang**

Supporting Information

Sperm miR-142-3p Reprogramming Mediates Paternal Pre-Pregnancy Caffeine Exposure-Induced Non-alcoholic Steatohepatitis in Male Offspring Rats

***Corresponding author:**
Hui Wang, PhD.,
School of Basic Medical Sciences, Wuhan University, Wuhan 430071, China
E-mail: wanghui19@whu.edu.cn.

Supplementary Results and Figures

1. PPCE induces hepatic histologic changes and lipid metabolic dysfunction in female offspring before and after birth

To further elucidate the role of PPCE in the development of NAFLD in the offspring, we also observed the effect of PPCE on the changes in hepatic lipid metabolism function in female offspring before and after birth. The results showed that PPCE could induce liver steatosis and significantly increase triglyceride levels in female offspring at GD20 (Figure S1 A, B). Meanwhile, the expressions of hepatic fatty acid synthesis and β-oxidation-related genes (*Srebp1*, *Fasn*, *Acc*, *Acly*, *Pparα*, and *Cpt1α*) were significantly increased in the PPCE group (Figure S1 C), while the expressions of inflammatory factors (*Tnf-α*, *Il-6*, and *Il-1β*) were not significantly changed (Figure S1 D). At PW32, a large amount of hepatocellular steatosis was observed in the liver of PPCE group accompanied by lipid accumulation and significantly elevated triglyceride content (Figure S1 E, F), but the degree of histological changes was significantly attenuated compared with the male offspring, and no obvious inflammatory infiltration and collagen fiber deposition were observed. Meanwhile, the expressions of hepatic fatty acid synthesis genes (such as *Srebp1*, *Fasn*, *Acc*, and *Acly*) were significantly increased and the expressions of β-oxidation genes (such as *Pparα* and *Cpt1α*) were decreased in PPCE group at PW32 (Figure S1 G), while there was no significant change in the expression levels of inflammatory factors (Figure

S1 H). Notably, no significant pathological changes were observed in the liver of F2 female offspring of PPCE (Figure S1I), and there were no significant changes in liver triglyceride content, fatty acid synthesis genes, fatty acid β-oxidation genes, or expression of inflammatory factors (Figure S1J-L). These results indicate that PPCE can cause changes in liver lipid metabolism and the development of NAFLD in female offspring, and there are significant gender differences.

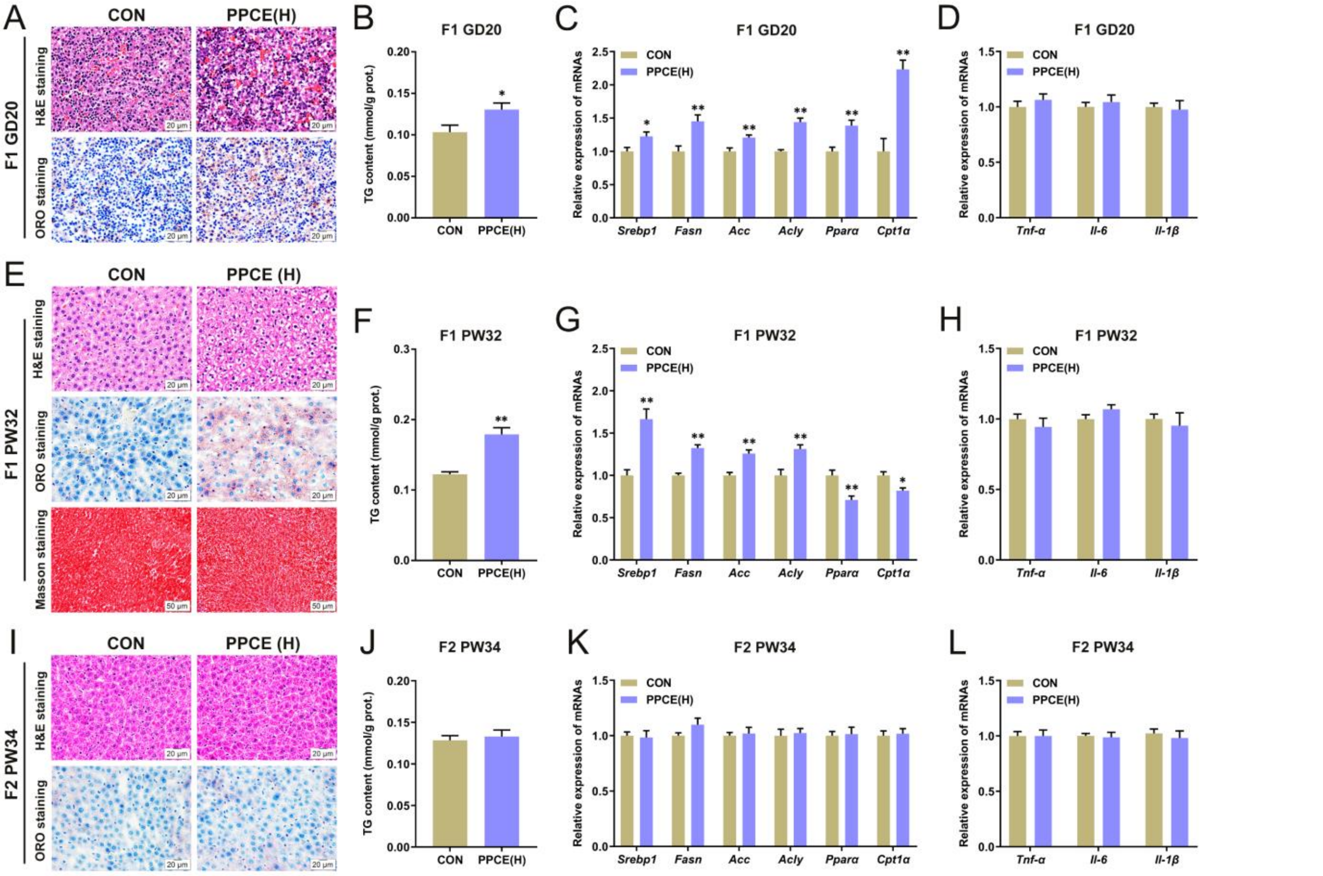

Figure S1. PPCE induces hepatic histologic changes and lipid metabolic dysfunction in female offspring before and after birth. (A) H&E and ORO staining at F1 GD20 (400×); (B) liver TG content at F1 GD20; (C) The mRNA expression of *Srebp1*, *Fasn*, *Acc*, *Acly*, *Pparα*, and *Cpt1α* at F1 GD20; (D) The mRNA expression of *Tnf-α*, *Il-6*, and *Il-1β* at GD20; (E) H&E, ORO, and Masson staining at F1 PW32 (200×, 400×); (F) liver TG content at F1 PW32;(G) The mRNA expression of *Srebp1*, *Fasn*, *Acc*, *Acly*, *Pparα*, and *Cpt1α* at F1 PW32; (H) The mRNA expression of *Tnf-α*, *Il-6*, and *Il-1β* at F1 PW32; (I) H&E and ORO staining at F2 PW34 (400×); (J) liver TG content at F2 PW34; (K) The mRNA expression of *Srebp1*, *Fasn*, *Acc*, *Acly*, *Pparα*, and *Cpt1α* at F1 F2 PW34; (L) The mRNA expression of *Tnf-α*, *Il-6*, and *Il-1β* at F2 PW34. Mean ± S.E.M., n = 10. $^{*}P < 0.05$, $^{**}P < 0.01$ *vs.* CON group.

2. PPCE causes activation of STING-mediated inflammatory response in the fetal liver of male offspring

Compared with the CON group, PPCE(H) significantly increased the fluorescence co-localization of STING and F4/80 in the fetal liver of male offspring (Figure S2A), while the mRNA expression levels of hepatic inflammatory factors (*Tnf-α*, *Il-6*, and *Il-1β*) were significantly increased (Figure S2B). It indicates that PPCE can induce activation of STING-mediated inflammatory response in the fetal liver of male offspring.

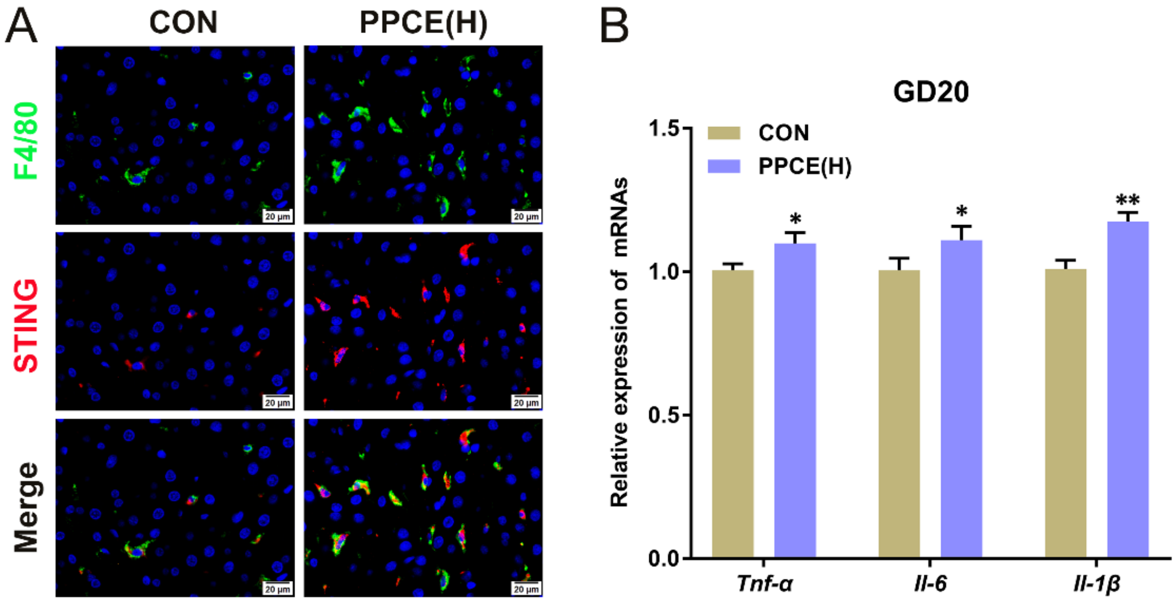

Figure S2. PPCE induces activation of STING-related inflammatory response in fetal liver of male offspring rats. (A) Typical immunofluorescence images and statistical analysis of fluorescence intensity for F4/80 and STING at GD20; (B) The mRNA expression of *Tnf-α*, *Il-6*, and *Il-1β* at GD20. Mean ± S.E.M., $n = 10$. $^{*}P < 0.05$, $^{**}P < 0.01$ *vs*. CON group.

3. Hepatic silencing of miR-142-3p leads to STING-mediated inflammatory activation

Compared with the CON group, miR-142-3p silencing (miR-142-3p$^{-/-}$) caused a significant increase in fluorescence co-localization of STING and F4/80 in mice liver (Figure S3A), while the protein expression levels of hepatic STING and p-NF-κB p65 (p-p65) were significantly increased (Figure S3B, C). It suggests that miR-142-3p$^{-/-}$ can cause activation of STING-mediated inflammatory response in mice liver.

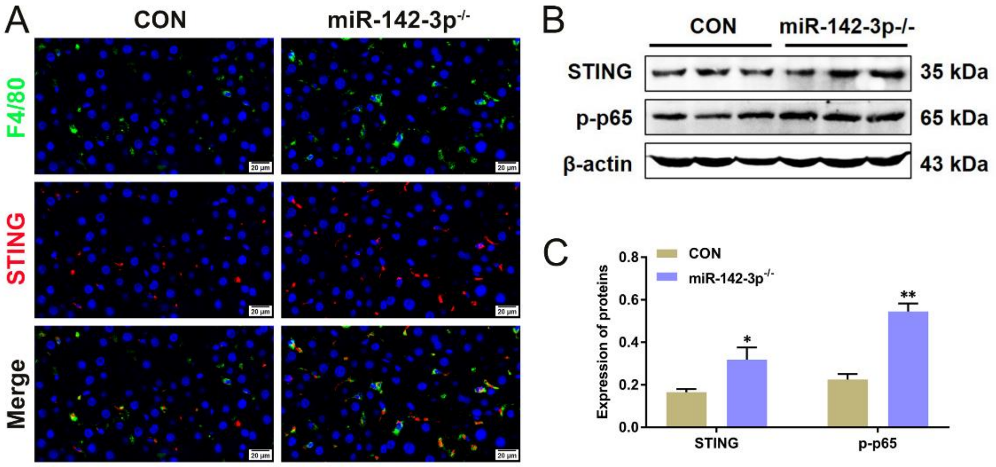

Figure S3. Hepatic silencing of miR-142-3p leads to STING-mediated inflammatory activation. (A) Typical immunofluorescence images and statistical analysis of fluorescence intensity for F4/80 and STING; (B, C) Representative immunoblots and quantitative analysis of STING and p-p65 protein. Mean ± S.E.M., n = 5. $^{*}P < 0.05$, $^{**}P < 0.01$ *vs*. CON group.

4. miR-142-3p regulates ACSL4 expression *in vitro*

ACSL4 mRNA and protein expression levels were examined after bone marrow mesenchymal stem cells (BMSCs) hepatoid differentiated cells treated with miR-142-3p mimics or miR-142-3p inhibitor. Our results showed that miR-142-3p inhibitor significantly elevated the mRNA level and protein expression of ACSL4 in BMSCs hepatoid differentiated cells, whereas miR-142-3p mimics significantly decreased the mRNA level and protein expression of ACSL4 (Figure S4).

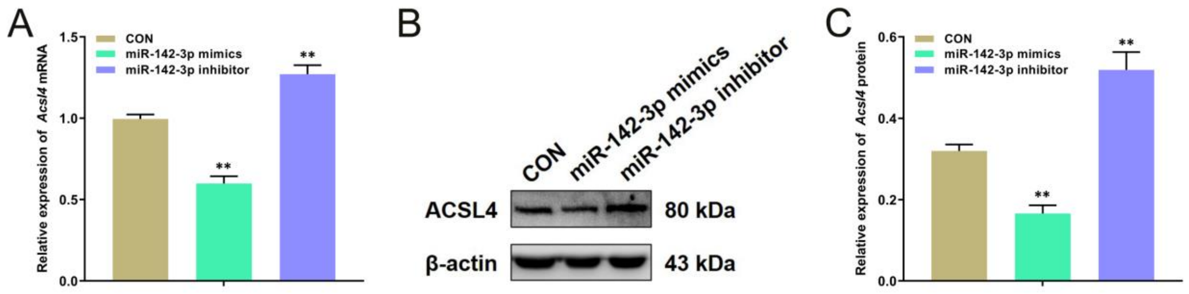

Figure S4. miR-142-3p regulates ACSL4 expression *in vitro*. (A) *Acsl4* mRNA expression after BMSCs hepatoid differentiated cells treated with miR-142-3p mimics or miR-142-3p inhibitor. (B, C) Representative immunoblots and quantitative analysis of ACSL4 protein. Mean ± S.E.M., n = 5. $^{**}P < 0.01$ *vs*. CON group.

5. Plasma cortisol concentration was significantly correlated with the methylation rate of miR-142-3p promoter region in sperm of men of reproductive age

In the adult male population, the sperm miR-142-3p promoter methylation rate was significantly higher in the high plasma cortisol group (plasma cortisol > 500 μg /L) than in the low plasma cortisol group (plasma cortisol ≤500 μg /L) (Figure S5A). The plasma cortisol level was positively correlated with the methylation level of miR-142-3p promoter region in sperm (Figure S5B). These results suggest that high glucocorticoid level is significantly associated with the methylation level of miR-142-3p promoter region in sperm. Further analysis showed that sperm miR-142-3p promoter methylation was not correlated with sperm count in adult males (Figure S5C), but was significantly positively correlated with sperm concentration and sperm malformation rate (Figure S5D, E). In conclusion, sperm miR-142-3p is expected to be a clinical early warning target for paternal NASH susceptibility. The plasma cortisol level is significantly correlated with the methylation level of miR-142-3p promoter region in sperm.

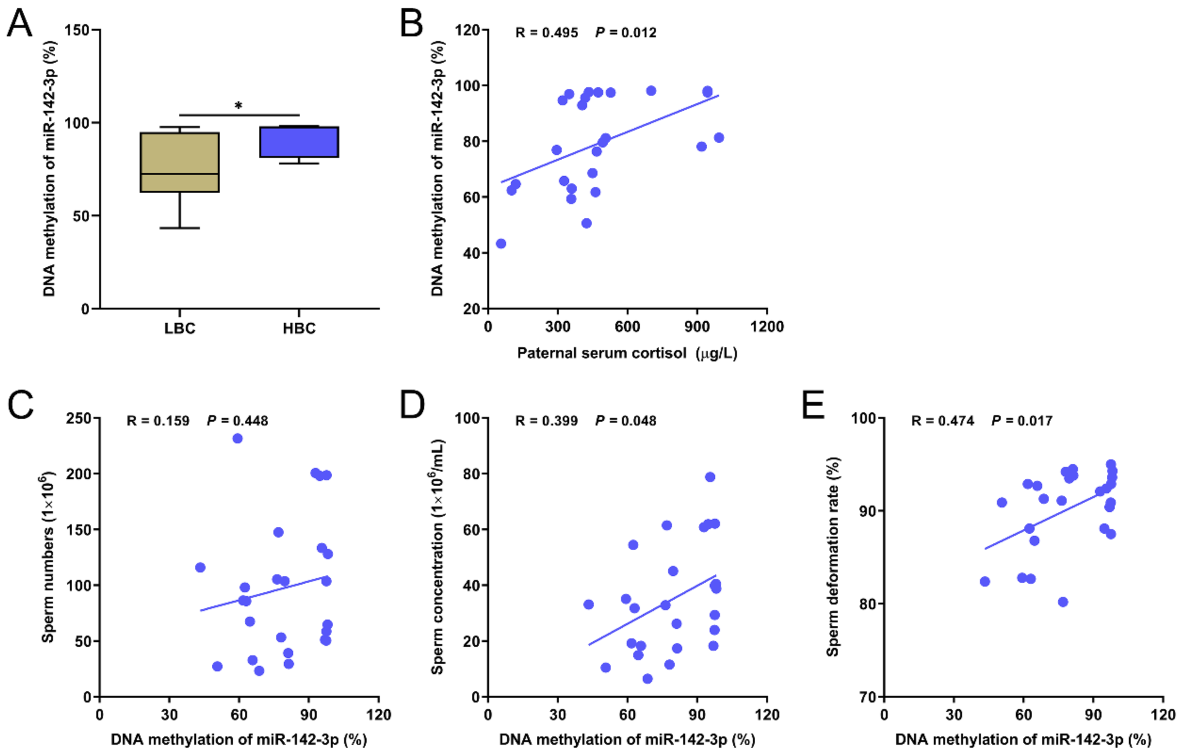

Figure S5. Human plasma cortisol concentration was significantly correlated with the methylation rate of miR-142-3p promoter region in sperm. (A) Sperm miR-142-3p promoter region methylation level of low blood cortisol (LBC) level and high blood corticosterone (HBC) level groups; (B) Correlation analysis of human serum cortisol level

and sperm miR-142-3p promoter region methylation level; (C) Correlation analysis of human sperm miR-142-3p promoter region methylation level and sperm numbers; (D) Correlation analysis of human sperm miR-142-3p promoter region methylation level and sperm concentration; (E) Correlation analysis of human sperm miR-142-3p promoter region methylation level and sperm deformation rate.

6. Paternal mixed exposure on altered lipid metabolism function in male offspring

To further identify the common mechanism of paternal NAFLD susceptibility by which miR-142-3p low expression mediates altered hepatic lipid metabolism function, we explored the impact of paternal mixed exposure (PME) to pre-pregnancy adverse exogenous (nicotine, ethanol, and caffeine) on altered hepatic lipid metabolism function in male offspring and the role of miR-142-3p low expression in it. Our results showed that compared with the CON group, PME resulted in significantly increased liver steatosis and triglyceride content in male offspring (Figure S6A, B), decreased miR-142-3p expression levels (Figure S6C), and significantly increased expression levels of fatty acid synthesis-related genes and decreased expression levels of β-oxidation-related genes (Figure S6D-G). Notably, the expression levels of miR-142-3p were significantly correlated with changes in the expression levels of lipid metabolism-related genes (*Srebp1*, *Fasn*, *Pparα*, and *Cpt1α*) in the fetal liver (Figure S6H-K). These results suggest that miR-142-3p low expression is closely associated with PME-induced altered lipid metabolism function in the fetal liver of male offspring.

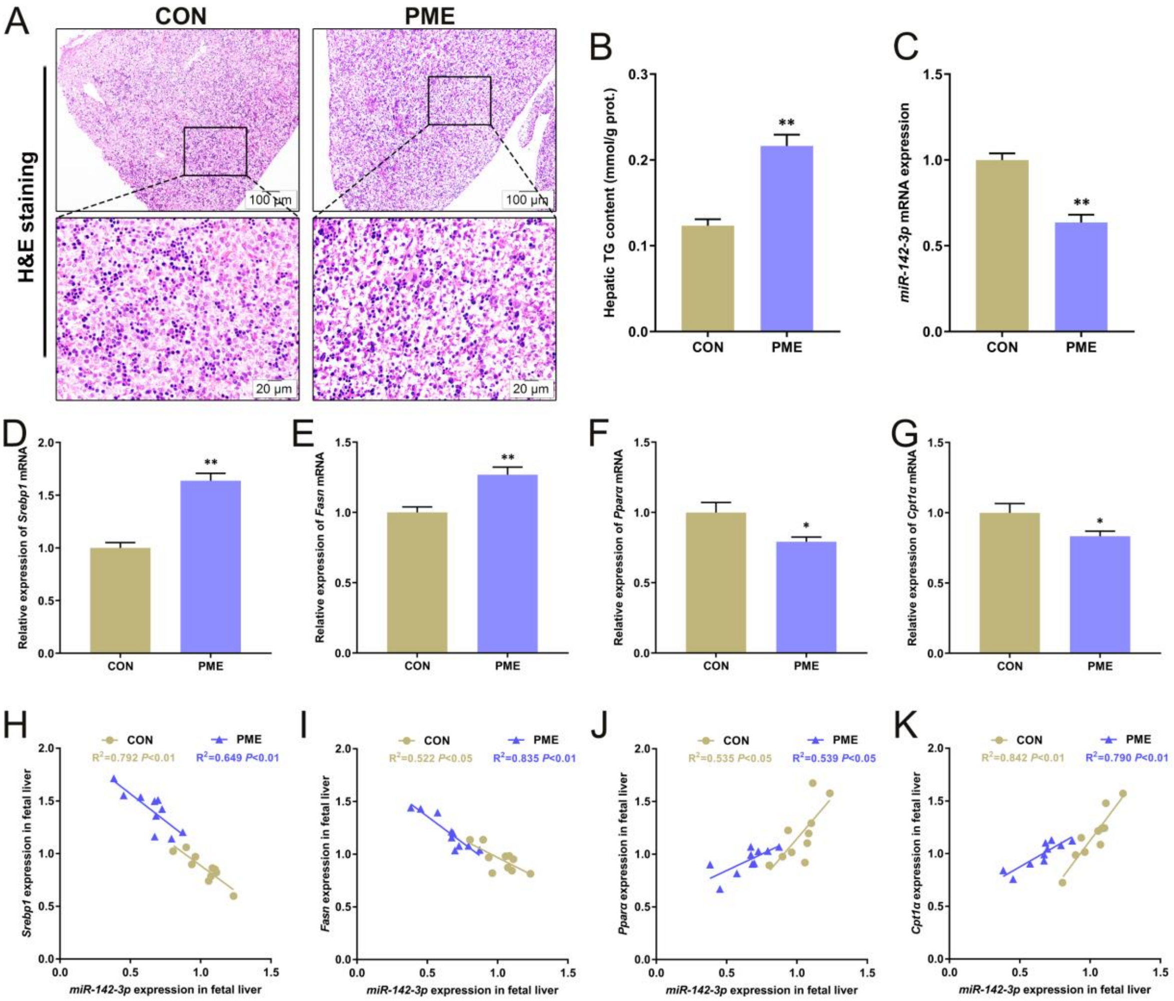

Figure S6. Effects of paternal mixed exogenous exposure on altered lipid metabolism function in male offspring. (A) H&E staining (100×, 400×); (B) liver TG content; (C-G) The mRNA expression of miR-142-3p, *Srebp1*, *Fasn*, *Pparα*, and *Cpt1α*; (H-K) Correlation analysis of miR-142-3p expression levels with those of Srebp1, Fasn, Pparα, and Cpt1α, respectively. Mean ± S.E.M., *n* = 10. $^{*}P < 0.05$, $^{**}P < 0.01$ *vs*. CON group.

Supplementary Tables

Table S1. Primer sequences of miR-142-3p mimics, miR-142-3p inhibitor, and miR-142-3p inhibitor-LV.

Genes	Sequences (5' to 3')
Rat-miR-142-3p mimics	Sense: UGUAGUGUUUCCUACUUUAUGGA<br>Antisense: CAUAAAGUAGGAAACACUACAUU
Rat-miR-142-3p mimics-NC	Sense: UUCUCCGAACGUGUCACGUTT

	Antisense: ACGUGACACGUUCGGAGAATT
Rat-miR-142-3p inhibitor	Sense: UCCAUAAAGUAGGAAACACUACA
Rat-miR-142-3p inhibitor-NC	Sense: CAGUACUUUUGUGUAGUACAA
Mus-miR-142-3p inhibitor-LV	Sense: TCCATAAAGTAGGAAACACTACA
Mus-miR-142-3p inhibitor-LV-NC	Sense: TTCTCCGAACGTGTCACGT

Table S2. Sequences of primers used in real-time quantitative PCR (RT-qPCR)

Genes	Sequences (5' to 3')	
Rat-GAPDH	F: GCAAGTTCAACG GCACAG	R: GCCAGTAGACTCCACGACA
Rat-Acsl4	F: AAGGAGAAGGGCAAAGAGA	R: GGTGGTTGTAGGAGGCTGA
Rat-Srebp1	F: CGCCCATCGGTTTAAGGACT	R: ACACTCGTTTCTTTCGGGCT
Rat-Fasn	F: TGGCTCAGCATGGCCGCTTC	R: CAGCTGTCGTTGGCCCCCTC
Rat-Acc	F: GAATCTCCTGGTGACAATGCTTATT	R: GGTCTTGCTGAGTTGGGTTAGCT
Rat-Acly	F: AGCCAAGATGTTCAGTAAAGCC	R: CATGTCTGGGTTGTTTATCGACT
Rat-Pparα	F: CACTGAACATCGAGTGTCGAA	R: AGCTTTAGCCGAATAGTTCGC
Rat-Cpt1α	F: ATCGCAAAGATCAGTCGGAC	R: AGCAGCACCTTCAGCGAGTA
Rat-Tnf-α	F: TACTGAACTTCGGGGTGATTGGTCC	R: CAGCCTTGTCCCTTGAAGAGAACC
Rat-Il-6	F: ACAGCCACTGCCTTCCCTAC	R: TTGCCATTGCACAACTCTTTTC
Rat-Il-1β	F: GGGATGATGACGACCTGC	R: CCACTTGTTGGCTTATGTT
Rat-miR-142-3p	F: TGCTGCTGTGTAGTGTTTCCTACT	R: TATGGTTGTTCACGACTCCTTCAC
Rat-U6	F: CTCGCTTCGGCAGCACA	R: GCGAGCACAGAATTAATACGAC
Mus-GAPDH	F: TCAATGAAGGGGTCGTTGAT	R: CGTCCCGTAGACAAAATGGT
Mus-Acsl4	F: ACTGGCGATATTGGAGAAT	R: CACATAGGACTGGTCACTT
Mus-Srebp1	F: TGACCCGGCTATTCCGTGA	R: CTGGGCTGAGCAATACAGTTC
Mus-Fasn	F: CCAAGCAGGCACACACAA	R: CACTCACACCCACCCAGA
Mus-Acc	F: GATGAACCATCTCCGTTGGC	R: GACCCAATTATGAATCGGGAGTG
Mus-Pparα	F: AGACACCCTCTCTCCAGCTT	R: TTCGCCGAAAGAAGCCCTTA
Mus-Cpt1α	F: GGCCATCTGTGGGAGTATGT	R: ACTGTAGCCTGGTGGGTTTG
Mus-Tnf-α	F: CAGGCGGTGCCTATGTCTC	R: CGATCACCCCGAAGTTCAGTAG
Mus-Il-6	F: TAGTCCTTCCTACCCCAATTTCC	R: TTGGTCCTTAGCCACTCCTTC
Mus-Il-1β	F: GAAATGCCACCTTTTGACAGTG	R: TGGATGCTCTCATCAGGACAG

Mus-miR-142-3p	F: GCCGCTGTAGTGTTTCCTACTT	R: GTGCAGGGTCCGAGGT
Mus-U6	F: TTGGTCTGATCTGGCACATATAC	R: AAAAATATGGAGCGCTTCACG
Rat-GAPDH	F: GTTACCAGGGCTGCCTTCTC	R: GGGTTTCCCGTTGATGACC
Rat-ND1	F: CTCCCTATTCGGAGCCCTAC	R: ATTTGTTTCTGCTAGGGTTG

Table S3. Antibodies used for western blot (WB) analysis and immunofluorescence (IF) analysis.

Protein	Antibody (catalog number)	Application	Dilution
ACC	Rabbit monoclonal (3676)	WB †	1:1000
ACSL4	Rabbit polyclonal (22401-1-AP)	WB; IF #	WB (1:1000); IF (1:100)
cGAS	Rabbit monoclonal (79978)	WB †	1:1000
CPT1α	Rabbit polyclonal (15184-AP)	WB; IF #	WB (1:1000); IF (1:100)
F4/80	Rabbit polyclonal (29414-1-AP)	IF #	1:100
FASN	Rabbit monoclonal (3180)	WB; IF †	WB (1:1000); IF (1:100)
p-p65	Rabbit monoclonal (3033)	WB †	1:1000
PPARα	Rabbit monoclonal (66826-1-Ig)	WB; IF #	WB (1:1000); IF (1:100)
SREBP1	Rabbit polyclonal (14088-1-AP)	WB; IF #	WB (1:1000); IF (1:100)
STING	Rabbit polyclonal (19851-1-AP)	WB; IF#	WB (1:1000); IF (1:100)
β-actin	Mouse monoclonal (66009-1-Ig)	WB #	1:10000

† Provided by Cell Signaling Technology Inc. (Danvers, MA).

Provided by Proteintech (Wuhan, China)

* Provided by ABclonal (Wuhan, China)
